# Gastric Point-of-Care Ultrasound in Acutely and Critically Ill Children (POCUS-ped): A Scoping Review

**DOI:** 10.3389/fped.2022.921863

**Published:** 2022-07-06

**Authors:** Frederic V. Valla, Lyvonne N. Tume, Corinne Jotterand Chaparro, Philip Arnold, Walid Alrayashi, Claire Morice, Tomasz Nabialek, Aymeric Rouchaud, Eloise Cercueil, Lionel Bouvet

**Affiliations:** ^1^Pediatric Intensive Care, Lyon University Children Hospital, Hospices Civils de Lyon, Lyon, France; ^2^School of Health and Society, University of Salford, Manchester, United Kingdom; ^3^Department of Nutrition and Dietetics, Geneva School of Health Sciences, HES-SO University of Applied Sciences and Arts Western Switzerland, Geneva, Switzerland; ^4^Department of Anaesthetics, Alder Hey Children's NHS Foundation Trust, Liverpool, United Kingdom; ^5^Department of Anesthesiology, Critical Care and Pain Medicine, Harvard Medical School, Boston Children's Hospital, Boston, MA, United States; ^6^Pediatric Intensive Care, Royal Children's Hospital, Melbourne, VIC, Australia; ^7^Pediatric Radiology Department, Lyon University Children Hospital, Hospices Civils de Lyon, Lyon, France; ^8^Department of Anesthesiology and Intensive Care, Lyon University Children Hospital, Hospices Civils de Lyon, Lyon, France

**Keywords:** pediatric intensive care, pediatric emergency, pediatric anesthesia, POCUS, nasogastric tube, foreign body, gastric insufflation, hypertrophic pyloric stenosis

## Abstract

**Introduction:**

Point-of-care ultrasound (POCUS) use is increasing in pediatric clinical settings. However, gastric POCUS is rarely used, despite its potential value in optimizing the diagnosis and management in several clinical scenarios (i.e., assessing gastric emptying and gastric volume/content, gastric foreign bodies, confirming nasogastric tube placement, and hypertrophic pyloric stenosis). This review aimed to assess how gastric POCUS may be used in acute and critically ill children.

**Materials and Methods:**

An international expert group was established, composed of pediatricians, pediatric intensivists, anesthesiologists, radiologists, nurses, and a methodologist. A scoping review was conducted with an aim to describe the use of gastric POCUS in pediatrics in acute and critical care settings. A literature search was conducted in three databases, to identify studies published between 1998 and 2022. Abstracts and relevant full texts were screened for eligibility, and data were extracted, according to the JBI methodology (Johanna Briggs Institute).

**Results:**

A total of 70 studies were included. Most studies (*n* = 47; 67%) were conducted to assess gastric emptying and gastric volume/contents. The studies assessed gastric volume, the impact of different feed types (breast milk, fortifiers, and thickeners) and feed administration modes on gastric emptying, and gastric volume/content prior to sedation or anesthesia or during surgery. Other studies described the use of gastric POCUS in foreign body ingestion (*n* = 6), nasogastric tube placement (*n* = 5), hypertrophic pyloric stenosis (*n* = 8), and gastric insufflation during mechanical ventilatory support (*n* = 4). POCUS was performed by neonatologists, anesthesiologists, emergency department physicians, and surgeons. Their learning curve was rapid, and the accuracy was high when compared to that of the ultrasound performed by radiologists (RADUS) or other gold standards (e.g., endoscopy, radiography, and MRI). No study conducted in critically ill children was found apart from that in neonatal intensive care in preterms.

**Discussion:**

Gastric POCUS appears useful and reliable in a variety of pediatric clinical settings. It may help optimize induction in emergency sedation/anesthesia, diagnose foreign bodies and hypertrophic pyloric stenosis, and assist in confirming nasogastric tube placement, avoiding delays in obtaining confirmatory examinations (RADUS, x-rays, etc.) and reducing radiation exposure. It may be useful in pediatric intensive care but requires further investigation.

## Introduction

Point-of-care ultrasound (POCUS) is the routine use of ultrasound, performed by non-radiology healthcare professionals at the bedside, to guide diagnosis and patient management. POCUS can address specific clinical questions, adding to the traditional physical examination. The wide availability of portable ultrasound devices and their ability to perform repeated non-invasive examinations have rapidly increased the use of POCUS in different clinical settings.

POCUS was initially used for cardiovascular assessment (1). More recently, the use of POCUS has been expanded to other organ systems, including the gastrointestinal system, in both adults and children (2, 3), but the use of gastric POCUS is currently not common.

The pediatric literature reports some data on the use of gastric POCUS in five main domains: (i) assessing gastric emptying and gastric volume/contents, (ii) gastric foreign body identification, (iii) confirming nasogastric (or orogastric) tube (NGT) position, (iv) hypertrophic pyloric stenosis (HPS) diagnosis, and (v) other indications (e.g., assessment of ventilation on gastric insufflation) (2, 4–12).

Gastric POCUS, performed by pediatric healthcare professionals (pediatricians, anesthesiologists, surgeons, emergency department (ED) physicians, specialist nurses) may be useful in various clinical settings and for different indications, but we currently lack a robust review of the literature to understand the role of gastric POCUS in children. We have conducted a scoping review (using a robust methodology) to answer this question, identifying the key domains that gastric POCUS has been utilized in pediatrics.

## Materials and Methods

An expert group was established, involving 10 members familiar with the use of gastric POCUS: two pediatricians, one clinical PICU nurse researcher, three pediatric anesthesiologists, two pediatric intensivists, one pediatric radiologist, and one methodologist. The scoping review was conducted in accordance with the JBI (Johanna Briggs Institute) methodology for scoping reviews (13). The study protocol was registered on Open Science Framework (OSF) on 28 February 2022 (doi: 10.17605/OSF.IO/P5BF9).

The review question (with five sub-questions corresponding to five aims) was discussed and defined as “What is the role of gastric POCUS in children?” (i) determining gastric emptying, (ii) assessing the placement of NGT, (iii) identifying ingested foreign bodies, (iv) diagnosing HPS, and (v) for other indications.

### Inclusion Criteria (Population, Concept, and Context)

Studies were considered if they were conducted in preterms, term neonates, and children up to 18 years. Prenatal studies were excluded. Studies had to directly assess the use of gastric POCUS but were excluded if gastric ultrasound was performed by radiologists (RADUS), rather than any bedside healthcare professionals (POCUS). The included studies could have been conducted in any pediatric setting [ED, pediatric intensive care (PICU), operating room (OR)] and must have answered the main question or a sub-question. Tumor diagnosis was out of the scope of the review as it was considered to require RADUS, rather than POCUS.

### Types of Studies/Sources

This scoping review considered both experimental and quasi-experimental study designs (randomized or non-randomized controlled trials, before and after studies, and interrupted time-series studies), analytical observational study designs (prospective and retrospective cohort studies, case–control studies, and analytical cross-sectional studies), descriptive observational study designs (case series, individual case reports, abstracts, and descriptive cross-sectional studies), systematic reviews, gray literature (abstracts from conferences and unpublished studies), qualitative studies, and text and opinion articles.

### Search Strategy

First, an initial limited search of MEDLINE (PubMed) was undertaken to identify studies on the topic. The text contained in the titles and abstracts and the MeSH terms used to index the articles were used by an academic librarian to develop a full search strategy for MEDLINE (PubMed; Supplementary Material 1). Search equations were further adapted for other databases and/or information source (Embase and Web of Science). Search equations were run in the three aforementioned databases. Filters were applied to search for studies published in English and French between 1998 and 2022. The reference lists of all included sources of evidence were also screened for additional studies.

### Study/Source of Evidence Selection

After duplicate removal, titles and abstracts were screened by two (or three in case of disagreement) independent reviewers (members of the expert group), following the inclusion criteria, on free online software (Rayyan QCRI) (14). Full texts of relevant studies/sources were retrieved and reviewed by one independent reviewer. They were excluded if they did not fulfill the inclusion criteria. The results were presented in a Preferred Reporting Items for Systematic Reviews and Meta-analyses extension for scoping review (PRISMA-ScR) flow diagram (15).

### Data Extraction

Data were extracted from included studies and consisted of study population characteristics, concept (aim of the study, gastric POCUS-related sub-question), context (setting, gastric POCUS operator), study designs, and relevant key findings.

### Data Analysis

Data are presented with a narrative summary, accompanied by tabulated and/or charted results. Where possible, quantitative summaries of extracted evidence are provided.

## Results

**Figure 1:**
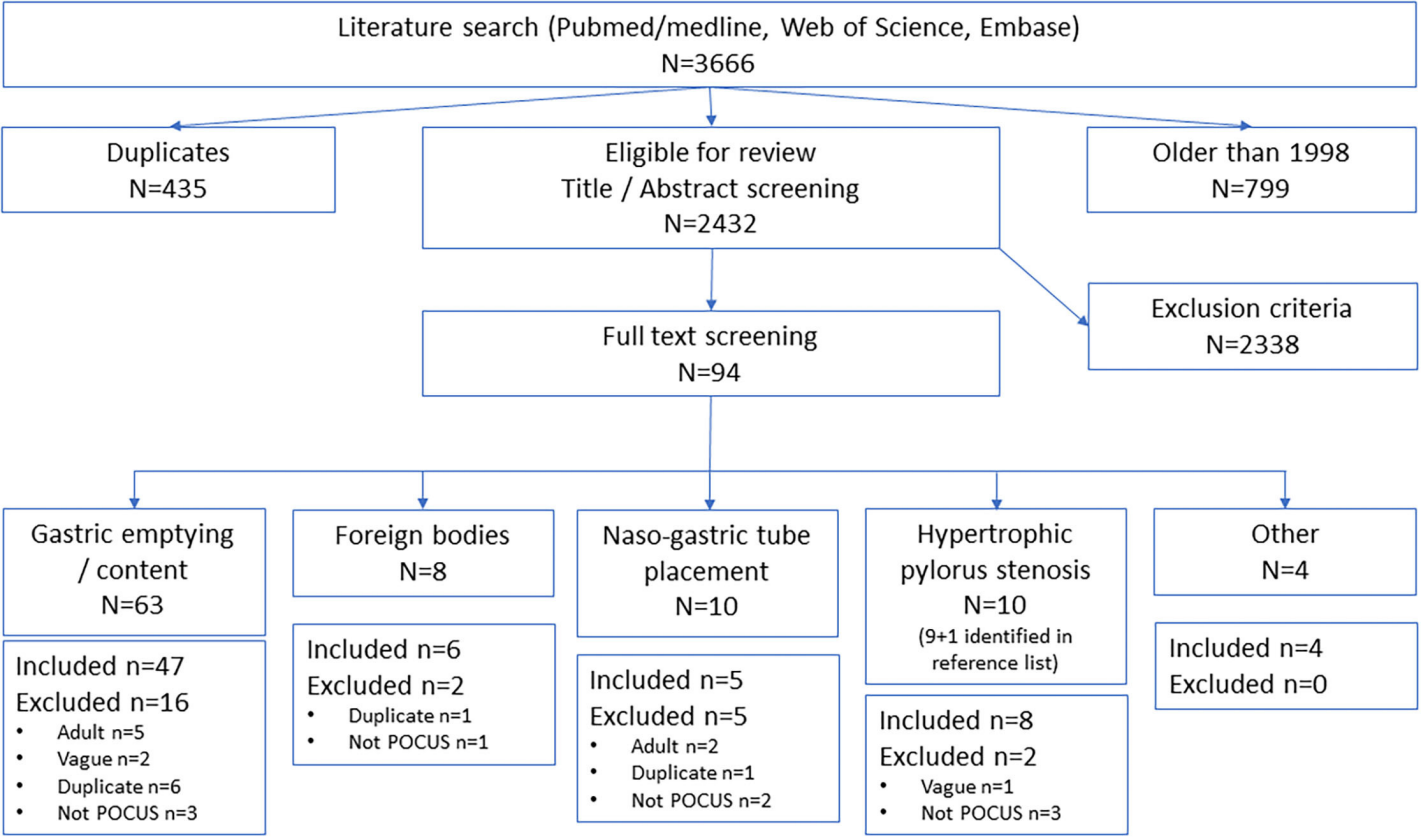
Prisma flow chart.

The literature search identified 3,666 articles, after removing duplicates and sources older than 1998, and 2,431 studies were eligible for screening (Figure 1). After abstract and full-text screening, a total of 69 articles were included, and one article was identified from another source.

**Tables 1**–**5** summarize the study characteristics and findings for each sub-questions.

**Figure 2:**
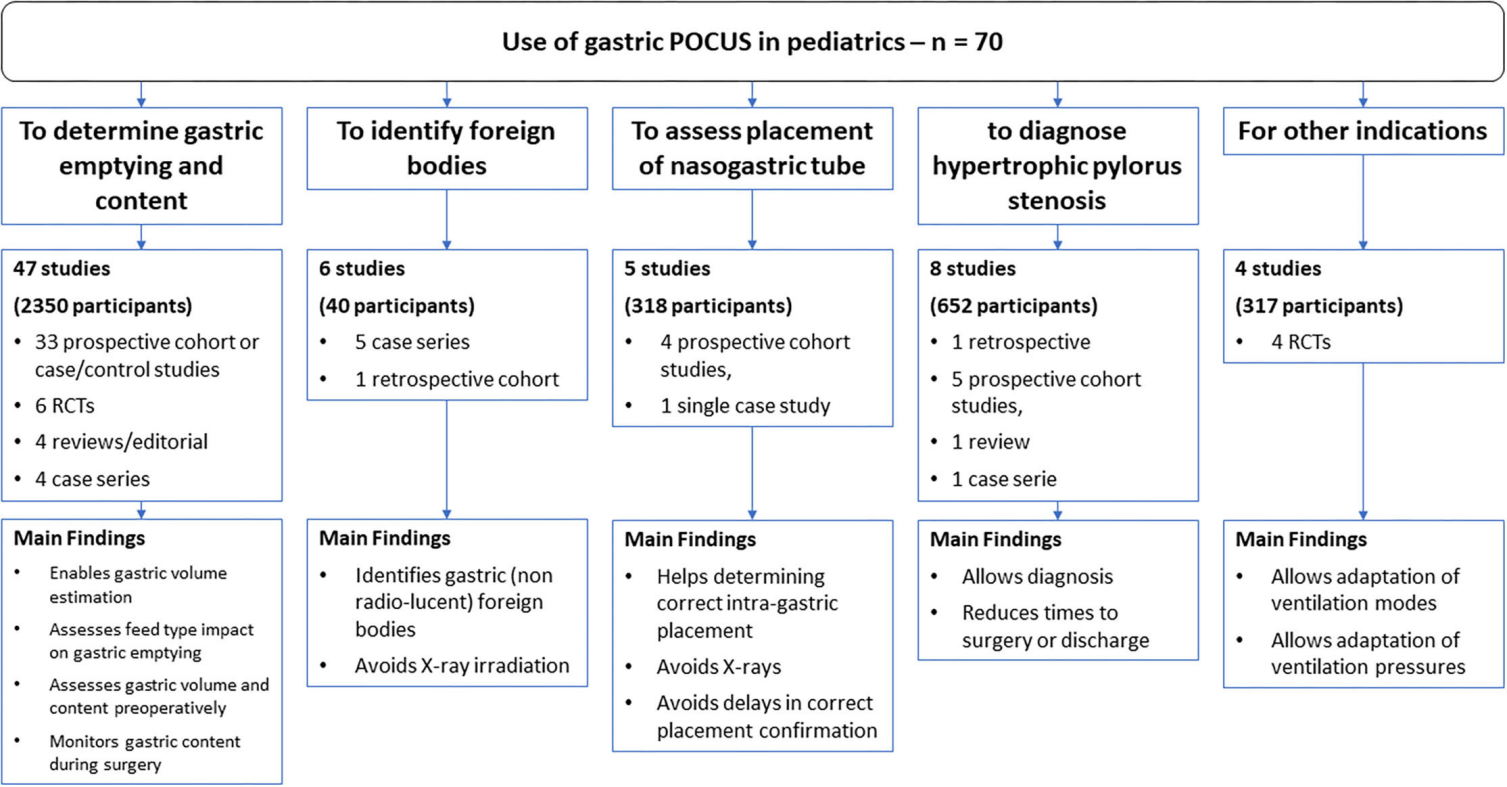
Literature search and main findings.

Most articles assessed gastric POCUS in one of the four main sub-questions, and the remaining four articles assessed its role in ventilatory support (see Figure 2).

## Gastric Emptying

Totally, 47 studies were included, in which gastric POCUS was performed by anesthesiologists and pediatricians (neonatologists) most of the time (Table 1) (2, 4, 6, 7, 16–58). Detailed results are available in Supplementary Material 2.

**Table 1:** Study characteristics and findings: gastric emptying and gastric content assessment.

**References**	**Study design**	**Patient characteristics**	**Gastric POCUS question**	**Intervention**	**Key findings**
Adler et al. (4)	Review	n/a	Gastric emptying	n/a	Review article that covered basics of POCUS and its utility
Anderson and Frykholm (16)	Observational cohort	55 children, ages 1–6 years old	Gastric emptying		Patients consumed yogurt or gruel (oatmeal); gastric POCUS was performed and found that one of the children had full stomach with consuming gruel. A light breakfast 4 h prior to induction may be considered, but there is need for further studies on safe limits for the volume ingested.
Azad et al. (17)	Prospective cohort	52 children in ED requiring sedation / anesthesia	Gastric emptying	Comparison of POCUS (ED physician) findings and fasting anamnesis to predict gastric content	Only 9 patients (17%) reported no food/liquid intake in past 2–6 h. The estimated sensitivity of gastric POCUS was 84% (95% CI 69–93%) and specificity was 22% (95% CI 4–60%) when compared to patient anamnesis as a gold standard. The positive likelihood ration (LR+) was 1.08 (95% CI 0.74–1.56) and the negative likelihood ration (LR–) was 0.73 (95% CI 0.17–3.09). This suggests gastric contents as seen on US correlated only modestly with patient history of ingestion.
Baldassarre et al. (18)	RCT	60 neonates (28–33 weeks; 700–1,750 gm)	Gastric emptying	Comparison of gastric emptying times between two formula solutions using POCUS. CSA measured as surrogate for volume.	No correlation between gastric emptying time and achievement of full enteral feeding was demonstrated for participants receiving an intact protein or extensively hydrolyzed study formula.
Bansal and Saini (19)	Prospective cohort; abstract only	70 total patients, 35 in each arm	Gastric emptying	Comparison of gastric cross-sectional area in children with 6 h of preoperative fasting vs. 2 h.	Poorly described abstract. No *p*-values to show that there were no major differences in the cross-sectional area. They also didn't describe position or how the measurements were acquired. Also, the conclusion that was reached (i.e., risk of aspiration) was not really measured in this study.
Beck et al. (20)	Prospective cohort	22 Neonates (mean age 35 weeks)	Gastric emptying	POCUS (performed by 1 NICU physician) compared gastric emptying time in preterm infants receiving formula vs. breastmilk	The study shows that the mean gastric emptying time after enteral feeding with breast milk and formula milk is <4 h in preterm infants.
Beck et al. (21)	Prospective observation	26 patients; children average age 11 years old	Gastric emptying	Patients were fasted, then given water / fruit juice and then gastric POCUS measured at 5, 15, 30, 45, 60 min	Gastric emptying time of children after intake up to 5 mL kg−1 clear fluids was <1 h in a clinical setting. These results support the more liberal fasting regimen favoring a 1-h fasting time and suggest 5 mL kg−1 as an upper limit for clear fluids (e.g., water, sugared water or tea or diluted fruit juice) from 2 to 1 h before induction of anesthesia in children.
Boretsky and Perlas (22)	Case report	2 cases	Gastric emptying	Report of a gastric POCUS (anesthetist) revealing full stomach after / before induction	Simple scans of the gastric antrum revealing a full stomach.
Bouvet et al. (2)	Prospective cohort	200 children; mean age 6.6 years old, 24 kg,	Gastric emptying	Children presenting for elective surgery were scanned (POCUS / anesthetist); used the Perlas grading system of 0–2 to assess volume	Mean fasting times for solids > 13 years, clears were 4 h Six (3%) children had a Grade 2 antrum (fluid content seen in both the supine and the right lateral decubitus positions). Two children had a gastric fluid volume >1.25 mL/kg. The prevalence of “at risk stomach” was 1% (95% confidence interval: 0.2–3.9%).
Charlesworth and Wiles (23)	Editorial / review	n/a	Gastric emptying	n/a	Reviewed the technique for gastric POCUS: Claimed “Although gastric ultrasound seems to be an easy technique to learn and perform, there remain several unanswered questions relating to clinical practice standards, image acquisition and analysis, clinical decision making and clinical governance.”
Desgranges et al. (24)	prospective cohort	66 patients undergoing ENT surgery; mean age 5	Gastric emptying	Gastric POCUS (anesthetist) was performed before induction and prior to extubation for ENT procedures to determine if the blood from surgery ends up in the stomach prior to extubation	Three providers conducted scans in both supine and lateral position. Cross sectional area was used to estimate the gastric volume. After elective ENT surgery, children are not at risk of a full stomach before tracheal extubation, and that pulmonary aspiration of blood that may occur after elective ENT surgery is probably not related to regurgitation of ingested blood from the stomach.
Du et al. (25)	Prospective RCT	48 children given apple juice, milk, or Ensure (protein containing milk substitute)	Gastric emptying	Patients were scanned (POCUS by anesthetist) at baseline, then every 30 min to assess volume for up to 6 h. Aim was to determine gastric emptying time from different liquid contents consumed.	Despite early differences, clearance from the stomach of apple juice, 2% milk or Ensure Clear is similar at the terminal phase, which is the period of greatest relevance to preoperative fasting recommendations. The stomach is essentially clear by 3–3.5 h for all three drinks studied. The differentiation between liquids in current guidelines is not supported by this study.
Elmetwally et al. (26)	RCT	30 fasting children; 20 supine, 10 semi-sitting;	Gastric emptying	Patients received 200 mL of fluid, Gastric POCUS (anesthetist) was conducted every 30 min until stomach was emptied; scan conducted by one person;	After 30 min of fluid ingestion, 40% of the semi-sitting group showed complete gastric emptying; whilst none of the children in the supine group showed complete gastric emptying after the same period
Evain et al. (27)	Prospective cohort	110 patients; undergoing urgent or semi-urgent fracture repairs. Mean age 10 years old	Gastric emptying	Gastric POCUS conducted in patients with fractures to determine whether or not higher risk gastric contents were present prior to induction	Children with an acute isolated extremity fracture, preoperative POCUS found gastric contents associated with a high-risk of pulmonary aspiration in more than one third of patients. Proximal limb fractures, preoperative opioid administration, and the absence of bowel sounds were associated with high-risk gastric contents. Conversely, an overnight rest between trauma and surgery was a protective factor.
Fabiani et al. (28)	prospective cohort	47 infants 1–12 months old with regurgitation	Gastric emptying	To evaluate the effects of thickeners on gastric emptying time; Two gastric POCUS scans (anesthetist) assessing gastric emptying time after receiving either a standard formula or a formula enriched with galactomannan.	The ingestion of a water-soluble fiber-enriched formula does not have any significant influence on the gastric emptying time of infants with frequent regurgitation or vomiting.
Frykholm et al. (29)	Review	Children	Gastric emptying	Guidelines on fasting prior to anesthesia	Gastric emptying may be studied with ultrasound imaging, which is increasingly reproducible and less invasive, although there may still be a measure of investigator variability
Fukunaga et al. (30)	Prospective cohort	44 children scheduled for planned surgery	Gastric emptying	POCUS (anesthetist). The volume of gastric contents was measured by aspirating through a nasogastric tube	CSA measured *via* POCUS was positively correlated with gastric volume (*r* = 0.56, *p* <0.0001).
Gagey et al. (31)	Prospective cohort	34 children in OR for HPS surgery	Gastric emptying	POCUS (anesthetist) of the antrum was performed before and after the aspiration of the gastric contents through a 10 French gastric tube. The stomach was defined as empty when no content was seen in both supine and RLD positions.	Nine (29%) had an “empty” stomach and 22 (71%) had a “full” stomach, during the first ultrasound examination of the antrum. The median (IQR) aspirated gastric volume was 2.2 (0.4–4.3) ml kg^−1^. In the nine infants with an “empty” stomach, the median (min –max) aspirated gastric fluid volume was 0.26 (0–0.59) ml kg^−1^, while the median (min—max) aspirated volume was 2.89 (0.86– 12.2) ml kg^−1^ in the 22 infants with a “full” stomach during the first ultrasound examination (*P* < 0.0001). After aspiration, 21/22 (95.5%) infants with a “full” stomach during the first ultrasound had an “empty” stomach during the second examination, and all the infants with an “empty” stomach during the first ultrasound also had an empty stomach during the second examination. Finally, non-rapid sequence induction was performed in 30/34 (88.2%) infants, while rapid sequence induction would have been performed for all infants, in the absence of any ultrasound examination of the gastric contents. A prediction linear model of gastric volume was built, with adjusted *R*^2^ value of 0.69.
Gagey et al. (32)	Prospective cohort	144 children in OR for emergency surgery	Gastric emptying	POCUS (anesthetist) in supine and RLD positions for assessment of gastric contents, using a 0–2 grading scale. A final induction plan was made prior and adapted after POCUS. Gastric contents were suctioned through a nasogastric tube; defined as above risk threshold for regurgitation and aspiration if there was clear fluid > 0.8 ml.kg^−1^, and/or the presence of thick fluid and/or solid particles.	Gastric ultrasound was feasible in 130 out of 143 (90%) of children and led to a change in the planned induction technique in 67 patients: 30 from routine to rapid sequence, and 37 from rapid sequence to routine. An appropriate induction technique was therefore performed in 85% of children, vs. 49% planned after preoperative clinical assessment alone (*p* < 0.00001). The results suggest that gastric ultrasound is a useful guide to the general anesthetic induction technique with respect to the risk of pulmonary aspiration, in comparison with pre-operative clinical assessment alone.
Gathwala et al. (33)	Case control	25 neonates (<37 weeks and <1,500 g) fully fed	Gastric emptying	The half gastric emptying time was measured using real time by POCUS (neonatologist) first on expressed breast milk (EBM) alone, then on EBM + Lactodex human milk formula (HMF). The antral CSA was measured before and after feed,	Mean half gastric emptying time was 24.00 ± 5.00 min on EBM and 24.40 ± 5.06 min on EBM + human milk fortifier (HMF). The same at 2nd assessment (15.2 ± 1.79 days), with EBM was 22.80 ± 4.58 min (EBM) vs. 23.60 ± 4.89 min when given EBM + HMF. These differences were not statistically significant.
Geddded et al. (34)	Prospective cohort (abstract)	20 term fully breastfed infants	Gastric emptying	POCUS (neonatologist)	Stomach volume was not associated with breast milk contents (leptin, protein, fat, casein, and lactose)
Kim et al. (35)	Prospective cohort	192 children planned for elective anesthesia	Gastric emptying	POCUS (anesthetist) was conducted using a qualitative grading system (0–2), and CSA measured in the supine position and RLD position. Quantification of gastric fluid volume by suctioning gastric content through a nasogastric tube.	Pearson correlation analysis showed that the gastric CSA in the supine (*P* <0.001; correlation coefficient: 0.667) and RLD (*P* <0.001; correlation coefficient: 0.845) positions and qualitative antral grade (*P* <0.001; correlation coefficient: 0.581) correlated with suctioned volume. We developed a predictive model: predicted volume (ml) = −3.7 + 6.5 × [right lateral decubitus cross-sectional area (cm^2^)] - 3.9 [supine cross-sectional area (cm^2^)] + 1.7 × grade (*P* <0.01). When comparing the predicted volume and suctioned volume, the mean bias was 0.01 ml/kg and the limit of agreement was −0.58 to 0.62 ml/kg
Lee et al. (36)	Prospective cohort	46 healthy newborns fed with formula	Gastric emptying	support or refute current preprocedural nil per oral (NPO) guidelines for neonates by determining gastric emptying times using POCUS (neonatologist) of the gastric antrum after formula feeding	Gastric emptying times ranged from 45 to 150 min and averaged 92.9 min (95% CI, 80.2–105.7 min; 99% CI, 76.0–109.8 min) in the overall study group. No significant differences were found in times to gastric emptying between male and female neonates [male: mean, 93.3 (95% CI, 82.4–104.2 min); female: mean, 92.6 (95% CI, 82.0–103.2 min); *P* = 0.930] or those delivered by vaginal vs. cesarean routes [vaginal: mean, 93.9 (95% CI, 81.7–106.1 min); cesarean: mean, 92.2 (95% CI, 82.5–101.9 min); *P* = 0.819].
Leviter et al. (6)	Prospective cohort	115 fasting children in ED prior to sedation	Gastric emptying	POCUS (ED physician) in supine and RLD positions, and interpreted as empty, liquid, or solid. Calculated the antral CSA; Gastric volume (mL/kg) was estimated [formula by (52)]	POCUS assessments took a median of 4 min (IQR = 3–5 min) to complete. One hundred and seven (93%) patients with evaluable images, of them, 74 patients 69% [95% confidence interval (CI) = 60–77%], were categorized as having a full stomach. Each hour of fasting was associated with lower odds (odds ratio = 0.79, 95% CI = 0.65–0) of a full stomach. Weighted kappa for inter-rater agreement was high = 0.74 (95% CI = 0.68–0.79).
Miller et al. (37)	Prospective cohort	103 children in ED with trauma requiring sedation procedures	Gastric emptying	POCUS (ED physician) was performed to evaluate gastric volume (qualitative + quantitative).	Air obstructing the posterior surface of the gastric antrum prevented measurement in 14. We observed a weak inverse correlation between fasting time (either liquid or solid) and estimated gastric volume (ρ = −0.33), with no significant difference based on type of intake (solids, ρ = 0.28; liquids, ρ = 0.22).
Miyazawa et al. (38)	Case control	39 infants with regurgitations	Gastric emptying	POCUS (unknown operator): Subjects were assigned randomly to three groups successively. HL-00, HL-350 and HL-450 (three types of infant formula that were identical except for the concentration of locust bean gum. Antral CSA was measured at 0, 30, 60, 90, 120, 150 and 180 min.	Antral cross-sectional areas at 60, 90, 120, and 150 min with HL-450, and at 60 min with HL-350, were greater than with HL-00. The median gastric emptying rate at 120 min with HL-450 (52.8%) was lower than with HL-00 (97.9%; *P* = 0.0019), while HL-350 (80.3%) and HL-00 did not differ significantly. The mean number of regurgitation episodes was significantly smaller when infants were fed with either HL-350 or HL- 450 than with HL-00. All stomachs were empty at 150 min.
Moser et al. (39)	Prospective cohort	100 children in OR for planned upper GI endoscopy	Gastric emptying	Following induction patients were scanned (POCUS anesthetist) in both supine and RLD positions. The endoscope aspirated stomach fluid content. Antral sonography was then completed in the supine and RLD positions.	Significant differences were found between pre-suctioned and post-suctioned CSA values in the RLD position. The cut-off CSAs of the empty antrum in the supine and RLD positions were 2.19 cm^2^ (sensitivity 75%, specificity 36%) and 3.07 cm^2^ (sensitivity 76%, specificity 67%), respectively. The RLD position produces the most sensitive and specific CSA cut-off value where an antral CSA of 3.07 cm^2^ in the RLD position presents with acceptable performance in the ability to discriminate an empty antrum in pediatric patients over 1 yr. of age. As age increases, the sensitivity and specificity of this test increases in the RLD position.
Munlemvo et al. (40)	Case report	4-year-old child prior to elective anesthesiology	Gastric emptying	POCUS (anesthetist)	Patient thought to be starving. Gastric POCUS showed food in the stomach and an empty stomach 2 h after.
Na et al. (41)	Prospective cohort	122 children scheduled for elective surgery	Gastric emptying	“Empty stomach” was defined as an empty antrum or a physiologic amount of gastric secretion (≤ 1.25 mL/kg) with gastric POCUS (anesthetist). Patients with solid contents or higher volumes of clear fluid were defined as not having an empty stomach.	For 95 patients who had followed the recommended fasting time, the median fasting time was 7 h for solids and 6 h for liquids, and 78 (82%) patients had an empty stomach. Conversely, seven of 27 patients (26%) who did not have an adequate fasting time had an empty stomach. The optimal cut-off value of fasting time to predict an empty stomach was 6.5 h based on a receiver operating characteristic (ROC) analysis (sensitivity = 0.767, specificity = 0.811). inter-rater agreement between the researcher and an expert reviewer was assessed, weighted kappa for inter-rater agreement was 0.75 (95% CI = 0.69–0.78)
Parekh et al. (42)	Case series	3 children planned for elective surgery	Gastric emptying	POCUS (anesthetist) prior to surgery	Two surgeries postponed because of gastric contents on POCUS. One empty stomach proceeded with a shorter starvation time.
Perella et al. (43)	Prospective cohort	24 preterms (28–35 Gestational weeks) on full enteral feeds	Gastric emptying	POCUS (anesthetist) Serial images of the antrum and stomach were recorded before commencement of the feed (0%), and during interruptions to feed delivery when 50, 75, and 100% of the total volume of the feed had been delivered. to acquire an image of the antrum CSA.	Spheroid calculation of stomach volume was the most reliable and valid measure of stomach volume. Fortified breast milk feeds were more echogenic than unfortified breast milk feeds. Residual stomach volumes (Median 2.12 mL, range 0.59–9.27 mL) were identified in 18 of 24 infants.
Perella et al. (44)	Prospective cohort	20 preterms (28–35 Gestational weeks) on full enteral feeds	Gastric emptying	POCUS (neonatologist) used to calculate gastric volumes and to rate echogenicity and intragastric curding for 20 infants. A total of 29 paired feeds of the same volume and composition were monitored prefeed and post feed	Our analyses of paired stomach volume measurements at matched time points indicate that when fed milk of the same volume and composition under similar conditions, stable preterm infants' serial gastric volume measurements are repeatable. Statistical comparison of paired measures at multiple time points instead of a single estimated gastric half-emptying time has provided more extensive information regarding gastric emptying rates over time than previously published. Of the paired stomach volumes measured, most (75%) were discrepant by <2 mL, with an intraindividual coefficient of variation of 14.2% immediately after the feed. These results indicate a high level of repeatability between sequential feeds.
Perella et al. (45)	Case control	25 preterms (28–35 Gestational weeks) on full enteral feeds	Gastric emptying	Stomach volumes of 25 paired unfortified and fortified feeds were monitored prefeed and post feed delivery. POCUS (by neonatologist) was used to calculate infant stomach volumes.	Breast milk composition influences gastric emptying in stable preterm infants, with feeds of higher casein concentration emptying faster during feeding than otherwise equivalent feeds, and FM 85 fortified mother milk emptying more slowly than unfortified mother milk.
Perella et al. (46)	prospective cohort	40 preterms (28–35 Gestational weeks) on full enteral feeds	Gastric emptying	Intra-individual comparisons were made for paired meals of 100% and 75% prescribed volume and identical composition of mother's own milk and pasteurized donor human milk. Serial stomach ultrasound images were used (POCUS by a neonatologist) to calculate gastric residual volumes (GRVs) and remaining meal proportions (% meal).	Gastric emptying was faster in the early postprandial period and slowed over time (*P* <0.001). Reduced volume meals had slower GE rates and lower GRV (*P* <0.001). Serial postprandial % meal was similar between reduced and full volume meals (*P* = 0.41). Higher milk casein concentration was associated with slower GE (*P* = 0.04). Complete gastric emptying (GRV = 0 ml) was more common in infants fed at 3 h intervals compared with those fed every 2 h (*P* = 0.002).
Schmitz et al. (47)	Prospective cohort	16 healthy children fasting overnight	Gastric emptying	Gastric content was examined before and at various instants after ingestion of 7 ml/kg diluted raspberry syrup. Gastric fluid volume (GFV) was determined by MRI and gastric CSA (POCUS Anesthetist) were measured in supine and RLD position.	Overall correlation between gastric CSA and GFV was poor to moderate in children, with the RLD position producing the most reliable results. Interpretation of isolated gastric CSA values could be misleading.
Schmitz et al. (48)	Prospective cohort	16 healthy children fasting overnight	Gastric emptying	Gastric content was examined by MRI and POCUS (anesthetist) in supine and RLD positions before, immediately after, and at various instants after ingesting 7 mL/kg) of standardized diluted raspberry syrup.	Gastric antral CSA was 221 ± 116, 218 ± 112, and 347 ± 188 mm^2^ for Supine position, elevated 45° supine, and RLD position, respectively. The best correlation between body weight corrected total gastric/gastric fluid volume (TGVw/GFVw) with gastric antral area was found for Right decubitus position (*R* = 0.79; *P* <0.01/*R* = 0.78; *P* <0.01). Bias and precision of calculated and measured GFVw was 0 ± 2.8 mL/kg Correlations between Gastric area and TGVw or GFVw in children are best in the RLD position, but not sufficient to predict GFVw with a given Gastric CSA.
Schmitz et al. (7)	Prospective cohort diagnostic test	18 healthy children	Gastric emptying	After Fasting overnight, children had a light breakfast and were investigated up to 4 or 6 h after. Gastric content was examined by MRI immediately followed by POCUS, first in the right lateral decubitus position (RLD) and subsequently in the supine position	72 POCUS examinations were completed. The corresponding 72 measurements using MRI volumetry ranged from 0.1 to 13.8 ml kg. The correlation between CSA and GCVw was superior for RLD, with *R* = 0.76 (*P* <0.001; 95%CI 0.76–1) and R = 0.57 (*P* <0.001; 95%CI 0.41–0.88) for the RLD and Supine approach, respectively. A simple linear regression formula for calculating gastric content volume per weight with CSA acquired in the superior RDL position was found, with *R*^2^ = 0.582, 95% limits of agreement ranging from +5 and−5 ml/kg (large limits +++). ROC plots revealed higher AUCs in the RLD position than in the Supine position for the diagnosis of gastric content volume > 1, > 1.5 and >2 ml/kg (89, 92, and 92% vs. 64, 68, and 66%)
Schmitz and Schmidt (49)	Editorial	NA	Gastric emptying	Comment on Bouvet et al. study	Before institutional resources are invested in training all anesthetists in GUS or before ultrasound specialists are engaged for selected cases, further research and discussion of the benefits of gastric POCUS should be undertaken.
Sethi et al. (50)	RCT	45 children scheduled for elective surgery	Gastric emptying	Four groups according to the test feed given 10 ml.kg-1 i.e., glucose (group I), low- fat milk (group II) and breast milk (group III) no food (group IV). The changes in Gastric CSA assessed by POCUS (anesthetist). After ensuring the attainment of basal values the stomach contents were aspirated.	Mean (SD) gastric emptying time in group I was 1.53 (0.25) h (range 1.00–1.75), group II 2.32 (0.31) h (range 1.75–2.75), and group III 2.43 (0.27) h (range 2.00–2.75). No children of group I and II were found to be “at risk” at 2 and 3 h, respectively, but 13.3% of group III children were labeled as “at risk” at 3 h. The incidence of “at risk” children in group IV was 33.3%. It was concluded that 3% fat milk or 17.5% glucose in a volume of 10 ml.kg^−1^ (maximum volume of 100 ml) can be given in children safely 3 and 2 h, respectively, before anesthesia.
Song et al. (51)	Prospective cohort	79 Children prior to elective surgery who had fasted for more than 8 h	Gastric emptying	To assess gastric volume in children using POCUS (anesthetist) before (8 h fasting) and 2 h after drinking carbohydrate fluids before surgery.	In all examinations, the gastric antrum was located successfully in the epigastric area. The mean (SD) of initial (fasting) and second (after drinking) US measurements were 2.09 (0.97) and 1.85 (0.94) cm^2^, respectively (*P* = 0.01; mean difference 0.24 cm^2^, 95% confidence interval 0.06–0.43). The fasting and after-drinking gastric volumes were both most strongly correlated with patients' height (*r* = 0.62 and *r* = 0.50, respectively, *P* <0.001). Patients' age (*r* = 0.56 and *r* = 0.45, respectively, *P* <0.001) and weight (*r* = 0.54 and *r* = 0.37, respectively, *P* <0.001) were also positively correlated with the fasting and after-drinking gastric volumes. However, there were no significant correlations between the volume of the carbohydrate drink and the fasting and after-drinking gastric volumes.
Spencer et al. (52)	Prospective cohort diagnostic test study	100 children undergoing elective gastric endoscopy	Gastric emptying	POCUS (anesthetist) measurement of the antral CSA in supine and RLD position was completed, and the antrum was designated as empty or non-empty. Gastric contents were endoscopically suctioned and measured.	Gastric antral CSA correlated with total gastric volume in both supine (*r* = 0.63) and RLD (*r* = 0.67) positions. A mathematical model incorporating RLD CSA and age (*R*^2^ = 0.60) was determined as the best-fit model to predict gastric volumes. Increasing gastric antral grade (0–2) was associated with increasing gastric fluid volume: Grade 0 (*n* = 54): corresponds to a median volume (IQR) /kg = 0.3 (0.2) ml/kg, Grade 1 (*n* = 37): 0.7 (0.4) ml/kg, and Grade 2 (*n* = 9): 1.4 (1) ml/kg (significant differences among the groups).
Spencer and Walker (53)	Prospective cohort	72 children undergoing elective gastric endoscopy	Gastric emptying	Comparison of two transducers used for POCUS (anesthetist) providing the best view was determined at the time of the ultrasound examination based on a combination of objective and subjective criteria	The best view of the antrum was achieved using a curvilinear transducer in 37 patients compared with 35 using a linear transducer. Age was significantly greater (mean rank 46.5 vs. 25.9, *P* <0.05) in patients for whom the best view was achieved using a curvilinear transducer. A similar association was noted with patient weight (mean rank 47.4 vs. 24.9, *P* <0.05).
Sümpelmann et al. (54)	Prospective cohort	35 healthy children	Gastric emptying	Gastric POCUS (by anesthetist) measures gastric antrum and calculates gastric volume as per Schitz formula after a normal breakfast	Measurement of gastric antral area (GAA) was possible in 95% of the cases. The first measurement was performed 51 +/- 31 (5–140) min and the second one 146 +/- 33 (40–220) min after breakfast. GAA correlated significantly with the fasting time (*r* = 0.69, *P* <0.0001, 95% CI 0.8–0.51). The first GAA after breakfast was significantly higher when compared to the second GAA before lunch [10.4 +/- 3.7 (1.7–17.8) vs. 5.5 +/- 2.6 (1.4–11.8) cm^2^; *P* <0.0001]. The calculated gastric volume (GV) correlated significantly with the fasting time (*r* = 0.69; *P* <0.0001, 95% CI 0.8–0.51). The calculated mean gastric emptying time was 236 min for GAA = 1 cm^2^ and 232 min for GFV = 0 mls
Taye et al. (55)	RCT	44 children planned for elective surgery, in the pre-operative room	Gastric emptying	POCUS was performed (anesthetist) to evaluate gastric contents and baseline Antrum CSA. Measurements were taken at baseline, immediately after ingestion of clear fluid (3 vs. 5 mL/kg) and after that at every 5 min till Antral CSA reached baseline level. Gastric emptying time and emptying half-time (t1/2) were determined.	In both groups, compared to baseline the antral cross-sectional area and gastric volume increased significantly following fluid ingestion and then decreased exponentially to reach baseline within 1-h. The median (IQR) (range) gastric emptying time (minutes) [35.0 (28.8, 40.0) (20.0–45.0) in group 3 and 40.0 (28.8, 45.0) (20.0–50.0) in group 5] and emptying half-time (minutes) [17.0 (15.7, 21.5) (14.4–24.0) in group 3 and 18.6 (16.0, 22.0) (15.1–23.8) in group 5] were comparable [median difference −5 (95% CI −7.8 to 2.1) and −1.5 (95% CI −2.3 to 1.0), respectively] (*p* = 0.16 and *p* = 0.44, respectively).
Yamaguchi et al. (56)	Case report	One child undergoing endoscopic gastrostomy	Gastric emptying	Gastric content described by POCUS (anesthetist) after 3 h fasting	Empty stomach. Only a small amount of the liquid was observed in right lateral decubitus position (RLD) and the cross-sectional area of the gastric antrum was 2.65 cm^2^, which is equal to 0.24–0.42 mL/kg. Endoscopy revealed the stomach was empty with limited aspiration of residual fluid
Yigit et al. (57)	Prospective cohort	20 newborns <1,500 g fed maternal milk with no fortifier	Gastric emptying	Antral CSA assessed by POCUS (neonatologist) after feeding with unfortified breast milk, half-fortified breast milk, and fully fortified breast milk	The average half-emptying time was 49 +23 min with breast milk, 54+ 29 min with half-fortified breast milk, and 65+ 36 min with fully fortified breast milk. The differences between feeding groups were not statistically significant.
Zhang et al. (58)	RCT	16 healthy children fasting from midnight	Gastric emptying	Children received 5 mL kg^−1^ of 5% glucose solution or preoperative CHI solution. All subjects underwent five POCUS (anesthetists) examinations at 10, 30, 60, 90, and 120 min	In the glucose solution group, the antral cross-sectional area and logarithms of gastric fluid volume returned to baseline at 30 min after ingestion. However, in the carbohydrate-rich drink group, the median [interquartile range; range] antral cross-sectional area [3.69 (2.64–5.15; 1.83–8.93) cm^2^ vs. 2.41 (2.10–2.96; 1.81–4.37) cm^2^, *P* <0.001] and mean (95% confidence interval) logarithms of gastric fluid volume [2.54 (2.30–2.79) mL vs. 2.12 (1.94–2.30) mL, *P* = 0.048] were still higher than at 60 min and returned to the baseline values at 90 min after ingestion, respectively. The degree of thirst was lower in the glucose solution group than that in the carbohydrate-rich drink group.

*POCUS, point-of-care ultrasound; RADUS, radiologist ultrasound; AUC, area under the curve; CSA, cross-sectional area; EBM, expressed breast milk; ED, emergency department; ENT, ear nose and throat; GAA, gastric antral area; GCV, gastric corrected volume; GFV, gastric fluid volume; GRV, gastric residual volume; HMF, human mother milk; MRI, magnetic resonance imaging; NICU, neonatal intensive care unit; OR, operating room; RCT, randomized controlled trial; RLD, right lateral decubitus; TGV, total gastric volume*.

These studies aimed (i) to assess gastric POCUS use to determine the gastric content volume; (ii) to assess gastric emptying from different amounts or types of breast milk (fortifier or not) or breakfast in infants or children; (iii) to determine the gastric contents/volume according to fasting duration, or in the setting of elective or emergency surgery in children; (iv) to assess whether ear nose and throat (ENT) surgery was associated with the change in the gastric content volume.

**Figure 3:**
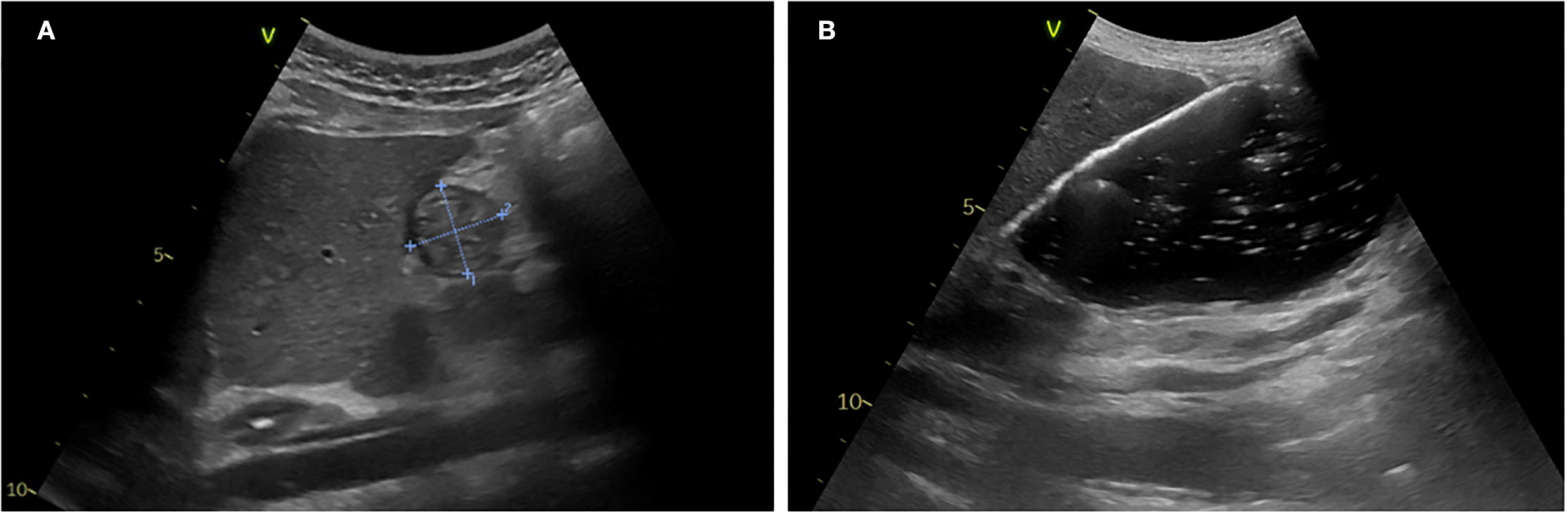
Gastric POCUS to assess gastric volume and content. **(A)** Empty stomach. **(B)** Full stomach (liquid content). Curvilinear low-frequency (2–5 MHz) or high-frequency linear transducers were used for examination (the former providing better scanning in older children). The gastric antrum was scanned in the epigastric sagittal plane, in the supine and/or in the right lateral decubitus position, for qualitative assessment and/or for the measurement of the antral cross-sectional area. In some studies, a longitudinal scan of the stomach was performed, allowing measurement of 3 diameters (anteroposterior, transverse, and longitudinal axes) for the calculation of the spheroid stomach volume (43, 44, 46). Repeated measurements minimize intra-rater variability. Gastric content volume was calculated in four studies using the mathematical model by (52) (*R*^2^ = 0.60), and in one study, it was calculated using the mathematical model by Schmitz et al. (7) (*R*^2^ = 0.582).

Figure 3 presents an example of gastric antrum measurements and details the gastric POCUS technique.

(i) Gastric ultrasound as a tool for estimating gastric content volume.

In most studies, the gastric antrum cross-sectional area (CSA) was measured, and gastric volume was calculated as per Spencer or Schmitz formulas (7, 47, 48, 52). In one research group, a longitudinal scan of the stomach was performed, allowing measurement of three diameters (anteroposterior, transverse, and longitudinal axes) for the calculation of the spheroid stomach volume (43, 44, 46).

In children older than 1 year, several studies reported a significant correlation between the antral CSA and gastric volume that was improved when ultrasound examination was performed in the right lateral decubitus position (7, 30, 35, 39, 47, 48, 52). Cut-off values for an antral CSA of 219 mm^2^ in the supine position and 307 mm^2^ in the right lateral decubitus position allowed discrimination between an empty or full stomach, with a sensitivity of 75% and a specificity of 36% in the supine position, and a sensitivity of 76% and a specificity of 67% in the right lateral decubitus position (39). Overall, three studies described mathematical models for predicting gastric contents volume compared to gastric endoscopy (52) or MRI (7) or gastric aspiration through the NGT (35), and one study developed a model for hypertrophic pyloric stenosis infants (31).

Spencer et al. (52) validated adult gastric contents. Perlas et al. made qualitative classification of gastric contents (77) to children aged 12 months−17 years; they reported that grade 0 (empty antrum in supine and right lateral decubitus (RLD) positions), grade 1 (fluid content seen in RLD only), and grade 2 (fluid content seen in both supine and RLD positions) were associated with gastric fluid volumes of <0.3, 0.3–1.5, and >1.5 ml/kg, respectively.

(ii) Feed type and amount impact on gastric emptying.

Repeated measurements of the antral cross-sectional area or spheroid stomach volume have been used to assess gastric emptying patterns in preterm neonates, term neonates, and children, not only to adapt to preoperative fasting rules but also as a surrogate of feed tolerance in term and preterm neonates and to identify factors associated with gastric emptying.

In term and preterm neonates, several studies investigated the impact of different feeding formula compositions on gastric emptying. These different feeds included formula thickening, feed formulas with various energy concentrations, fortified or non-fortified expressed breast milk, hydrolyzed protein feeds, and the child's position and feed volume, with inconsistent results among the studies (18, 28, 33, 38, 44–46, 57). In preterms, Beck et al. found the mean gastric emptying time of breast milk was <4 h, and Lee et al. reported a mean gastric emptying time after formula feeding of 93 min, ranging from 45 to 150 min (20, 36).

In children, several studies assessed gastric emptying times of different meals, formulas, and clear fluids. The gastric emptying time was <4 h after low-fat milk, breast milk, and a light breakfast (16, 50, 54, 78); <1 h after clear fluids (21); and <90 min after a carbohydrate-rich drink (58). Song et al. reported gastric fluid volume 2 h after a carbohydrate drink were lower than that after prolonged fasting (51), while a pilot study reported that gastric emptying of apple juice, 2% milk, or a high-protein drink was >4 h in children aged 8–14 years (25). Furthermore, fluid volume (3 vs. 5 ml/kg) did not affect gastric emptying in 44 children older than 6 years (55), and gastric emptying of clear fluids was enhanced when children were positioned in a semi-seated position (26).

(iii) Preoperative gastric volume assessment.

Several studies assessed the percentage of full stomach in elective and emergency anesthesia/surgery. In fasting children planned for elective surgery, gastric content volume was low (79) and the percentage of “stomachs at risk of aspiration” was 1% (2), but in the emergency setting, Gagey et al. and Evain et al. reported the incidence of full stomach to be between 51 and 37%, respectively (27, 31, 32). In children, fasting for more than 6 h admitted to the ED and requiring procedural sedation, 18–69% of these had a full stomach (6, 37, 41). Gagey et al. reported that an ultrasound-guided anesthetic strategy led to an 85% appropriate induction sequence technique compared to 49% after clinical assessment alone in non-elective children (31). In addition, ultrasound-monitored gastric aspiration allowed the performance of a non-rapid induction sequence in 88% of 34 infants scheduled for pyloromyotomy (31, 32).

(iv) Monitoring of gastric content during surgery.

Desgranges et al. reported no significant changes assessed by gastric POCUS in gastric volume occurred during ear nose and throat (ENT) surgery. This surgery bleeding did not increase the risk of postoperative aspiration (24).

## Foreign Bodies

**Figure 4:**
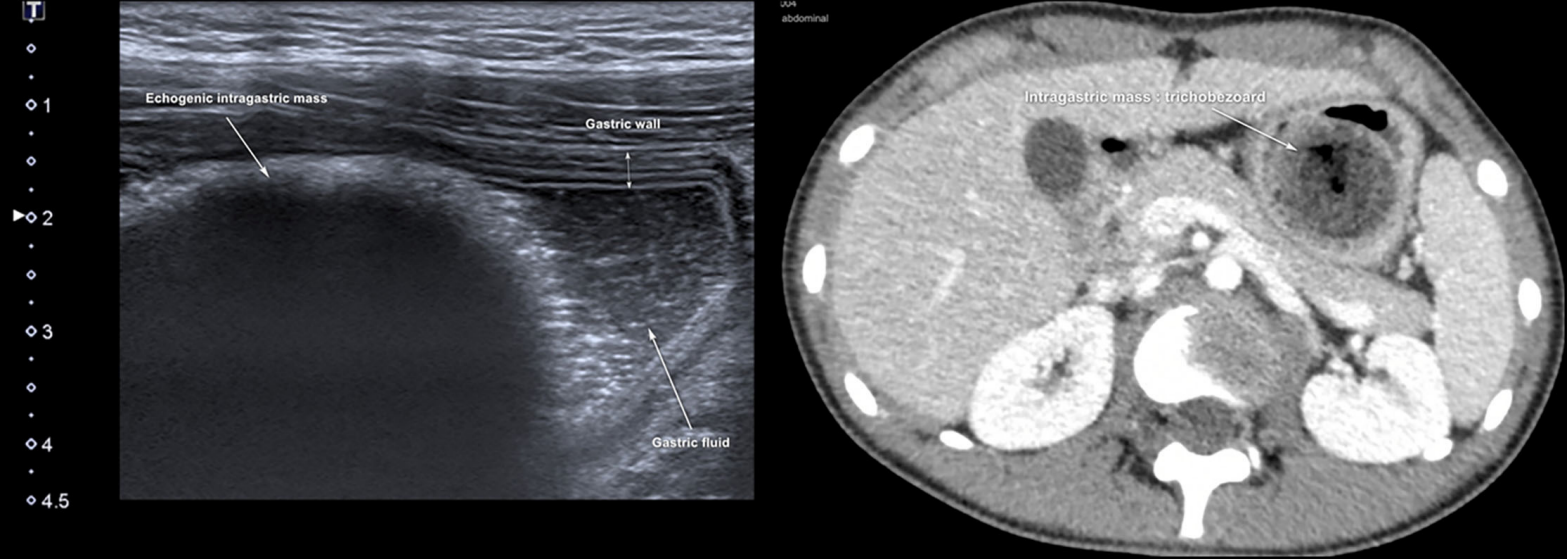
Intragastric foreign body. Large hyperechoic mass within the stomach may suggest the diagnosis of an intragastric foreign body (e.g., voluminous trichobezoar). In an uncertain diagnosis, and before surgery, an abdomino-pelvic CT scan should be performed to examine the extension of the foreign body. Gastric POCUS techniques found in the literature: various ultrasound probes have been used (high- or low-frequency linear or curvilinear transducers) and the child was positioned in a supine and/or a right lateral decubitus to enhance the quality scanning of the thoracic and epigastric areas. Liquid filling may help in visualizing the foreign body.

Totally, six studies (10, 59–63) were identified, and all were conducted in the pediatric ED (Table 2). They reported the use of gastric POCUS to diagnose foreign body ingestion and detect the presence of foreign bodies in the stomach and follow up in relation to gastric clearance. They noted only short training of ED physicians was required to perform gastric POCUS. Figure 4 presents an example of an intragastric trichobezoar (hair or wool solid mass) and details the gastric POCUS technique. These studies showed that gastric POCUS could be used to confirm foreign body ingestion, compared to x-ray. These findings may reduce the need for routine x-rays, which are not valuable in the case of both radiolucent and non-radiolucent masses. Gastric water filling may further help foreign body visualization.

**Table 2:** Study characteristics and findings: foreign body diagnosis.

**References**	**Study design**	**Patient characteristics**	**Gastric POCUS question**	**Intervention**	**Key findings**
Buonsenso et al. (10)	Case series	Eight children in ED	Foreign body	Five ED physicians who participated to a 2-day POCUS workshop	Foreign bodies were visualized on ultrasound as a hyperechoic structure with back acoustic shadowing and all were confirmed by X-ray or endoscopy.
Horowitz et al. (59)	Case series	Three children in ED	Foreign body	POCUS performed by a ED physician who was a trained Emergency Ultrasound fellowship.	Two of the three FB were confirmed with standard radiographs, one was not identified radiographically but was passed in the stool. All three objects were initially found in the stomach using POCUS as hyperechoic structure and reverberation artifact.
Jecković et al. (60)	Retrospective cohort	18 children in ED	Foreign body	Ultrasound examination of water-filled stomach performed by a ED physician	The gastric foreign bodies (eight coins, five button batteries, domino, lollipop stick, hairclip, screw nut, and small plastic cylinders) were confirmed by ultrasound even those radiolucent. US depicts FBs of any nature as hyperechoic structures, with sometimes an acoustic shadowing (depends on the composition of the FB and the incidence of the beam).
Salmon and Doniger (61)	Case series	Two children in ED	Foreign body	POCUS performed by an ED physician and confirmed by X-ray.	The examination was performed in 2 positions. At the thoracic inlet: the patient is placed in the supine position, and the probe is placed transversely anterior neck overlying the cricoid cartilage. The coin foreign body appears hyperechoic in the left paratracheal space. Stomach/epigastric area: the patient is placed in the supine or in the right lateral decubitus position, probe at the subxiphoid region. Water-filled the stomach if it's not well-visualized. FB appears as a hyperechoic lesion in the stomach, with posterior acoustic shadowing and comet tail artifact.
Spina et al. (62)	Case report	4 years old asymptomatic patient in ED	Foreign body	FB was assessed by X-ray and compared with abdominal US (ED physician) after drinking 300 ml of tea	US images of the upper abdomen show the hyperechoic FB inside the liquid filled stomach as an hyperechoic lesion with an acoustic shadow and comet tail artifact inside the stomach.
Yamamoto et al. (63)	Case report	5 years old patient in ED	Foreign body	Ultrasound examination by an attending ED physician, then confirmed by abdominal X-ray.	US examination is performed in the upright and slightly forward tilting position. Marble appears as a hyperechoic semicircular structure in the stomach with posterior acoustic shadowing and reverberation artifact noted posterior to the midline of the structure.

*POCUS, point-of-care ultrasound; RADUS, radiologist ultrasound; CSA, cross-sectional area; ED, emergency department; RCT, randomized controlled trial; FB, foreign body*.

## Nasogastric (or Orogastric) Tube Placement

**Figure 5:**
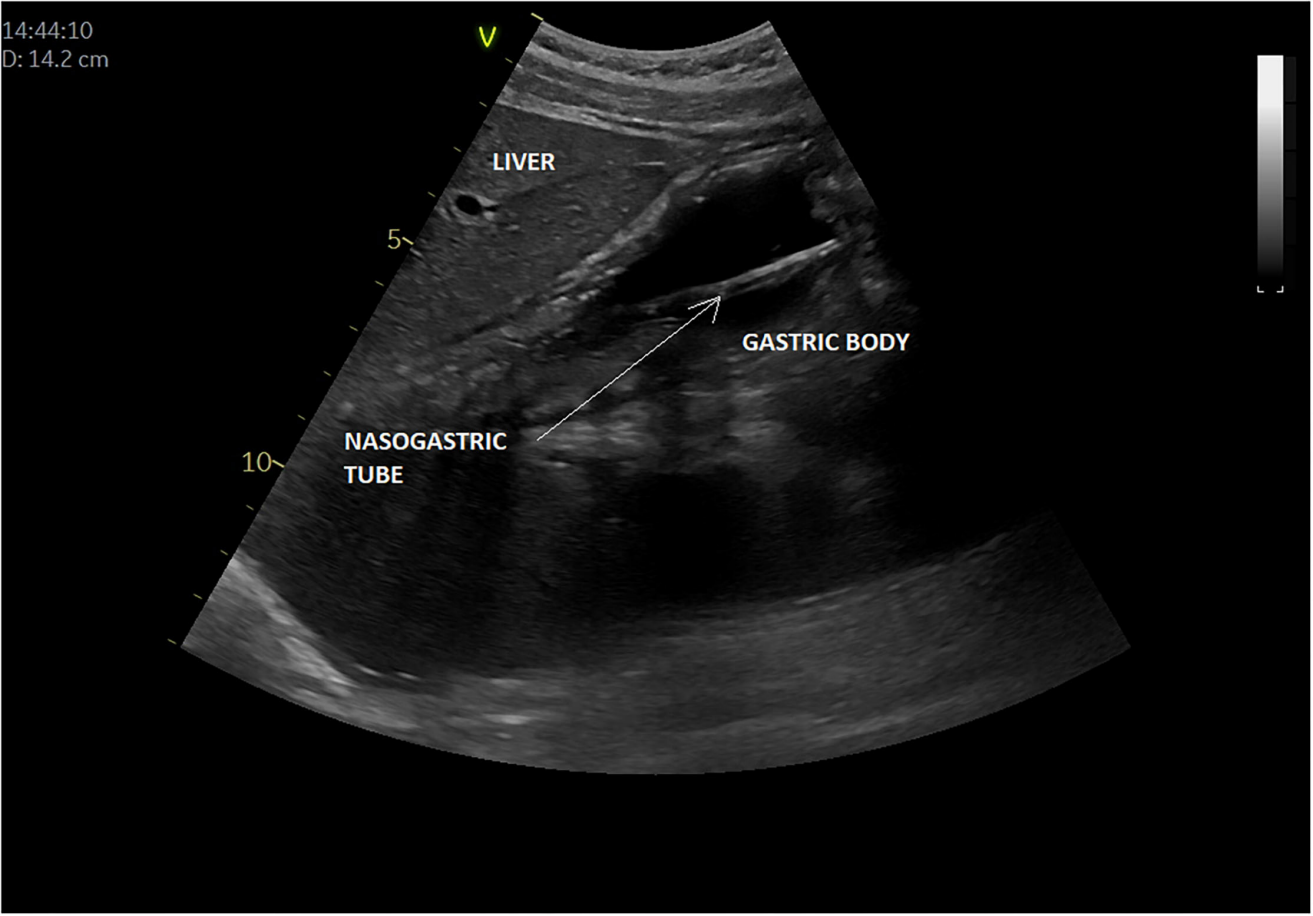
Intragastric nasogastric tube (or orogastric tube). The gastric POCUS technique found in the literature: The NGT was visualized using the curvilinear transducer or the phased transducer (with the iScan feature to optimize the view). Probe frequency was adapted to the size of the patient. The child was positioned in a dorsal decubitus position. The transducer was positioned in the middle of the epigastric region, allowing for visualization of the tube passing through the cardia and entering the gastric area. Then the transducer was positioned in the upper right quadrant toward the duodenum, to verify whether the tube was entering the pylorus. The correct position of the NGT corresponded to a hyperechogenic line passing through the cardia with its length continuing within the gastric area but not entering the pylorus. Otherwise, the transducer was placed transversely over the xiphisternum and was fanned downward and aimed toward the left upper quadrant to visualize the gastric body through the left lobe of the liver. Then, sagittal and transverse sweeps were performed over the epigastric area. If the NGT was not identified, the transducer was placed over the left flank in the sagittal position using the spleen as a window. The study was considered positive when the NGT could be visualized in the stomach as two parallel hyperechoic lines.

In all, five articles were included (8, 9, 64–66) (Table 3). They all focused on the accuracy of confirmation of the NGT position by bedside ultrasound in children and preterm neonates; two studies involved only newborns and preterms (8, 64), the others involved children older than 16 years (9, 65, 66); two studies compared ultrasound placements to abdominal x-ray (as gold standard), and the other ultrasound placements to “standard confirmation techniques.” POCUS was performed by a pediatrician, an ED physician, or a neonatologist, with only two of the studies being blinded. Figure 5 presents an example of NGT in the stomach and details the gastric POCUS technique.

**Table 3:** Study characteristics and findings: naso-(oro)gastric tube placement.

**References**	**Study design**	**Patient characteristics**	**Gastric POCUS question**	**Intervention**	**Key findings**
Atalay et al. (64)	Prospective cohort, diagnostic test	102 newborns in NICU	Naso-Gastric tube placement	NGT position accuracy assessed by POCUS (neonatologists) was compared with abdominal X-ray	Sensitivity reported as 92.2% and PPV as 100%. 7.8% (4) location of NGT could not be determined by US.
Choi et al. (65)	Prospective observational diagnostic test	30 children (stratified 3 age groups) requiring NGT placement	Naso-Gastric tube placement	NGT insertion and position assessed by US by pediatrician (unblinded) and NGT position confirmed by “usual procedures”	At the gastric antrum level, US views showing successful NGT placement was limited to 15 of 29 patients [52% (95% CI: 33–71%), *P* = 1.0]. Subgroup analysis showed that successful visualization of tube placement in the stomach ranged from 40% (7–18 years) to 70% (3–6 years). Eighty percent of air boluses injected were visualized
Claiborne et al. (9)	Prospective observational diagnostic test	26 children mean age 2.6 years in ED	Naso-Gastric tube placement	NGT position accuracy confirmed by x-ray was assessed by blinded ED physicians	Sensitivity of ultrasound for detecting a properly placed tube was 88% (95% confidence interval, 70.0–97.6%). 3/26 NGTs could not be visualized by US
Dias et al. (8)	Prospective double blind observational study	159 spontaneously breathing newborns in NICU	Naso-Gastric tube placement	NGT placed by nurses, then position confirmed by US (by trained neonatologist blinded) then compared to X-Ray	The tubes were correctly positioned in 157 cases (98.7%), according to radiological images, and in 156 cases (98.1%), according to ultrasound. The sensitivity analysis was 0.98 and the positive predictive value was 0.99
Mori et al. (66)	Case report	One 3 year old boy with difficulty placing NGT in ED	Naso-Gastric tube placement	NGT placed by US guidance and tube position in stomach confirmed	The entry of the NGT tip into the gastric cardia was confirmed on the subxiphoid longitudinal view. A chest radiograph confirmed the presence of the NGT in the stomach.

*POCUS, point-of-care ultrasound; RADUS, radiologist ultrasound; US, ultrasound; NICU, neonatal intensive care unit; NGT, naso-(oro-)gastric tube*.

Of the three studies reporting sensitivity of POCUS to confirm the GT position, this ranged from 88% (95% confidence interval, 70.0–97.6%) to 98.1% (8, 9, 64). However, there was considerable variability between the studies regarding POCUS being unable to determine the position of the NGT: 7.8% (Atalay et al.), 48% (Choi et al.), but in <2% (Dias et al.). In the Choi study (65), broken down by age of the child, at the gastric antrum level, POCUS showing successful NGT placement was limited to 15 of 29 patients [52% (95% CI: 33–71%)]. A subgroup analysis showed that tube placement in the stomach was visualized in four of nine patients [44% (95% CI: 14–79%)] between 0 and 2 years old, 7/10 [70% (95% CI: 35–93%)] aged 3–6 years and in four of 10 patients [40% (95% CI: 12–74%)] > 6 years old. The use of an air bolus (1 ml/kg) improved NGT visibility in some children.

## Hypertrophic Pyloric Stenosis (HPS)

**Figure 6:**
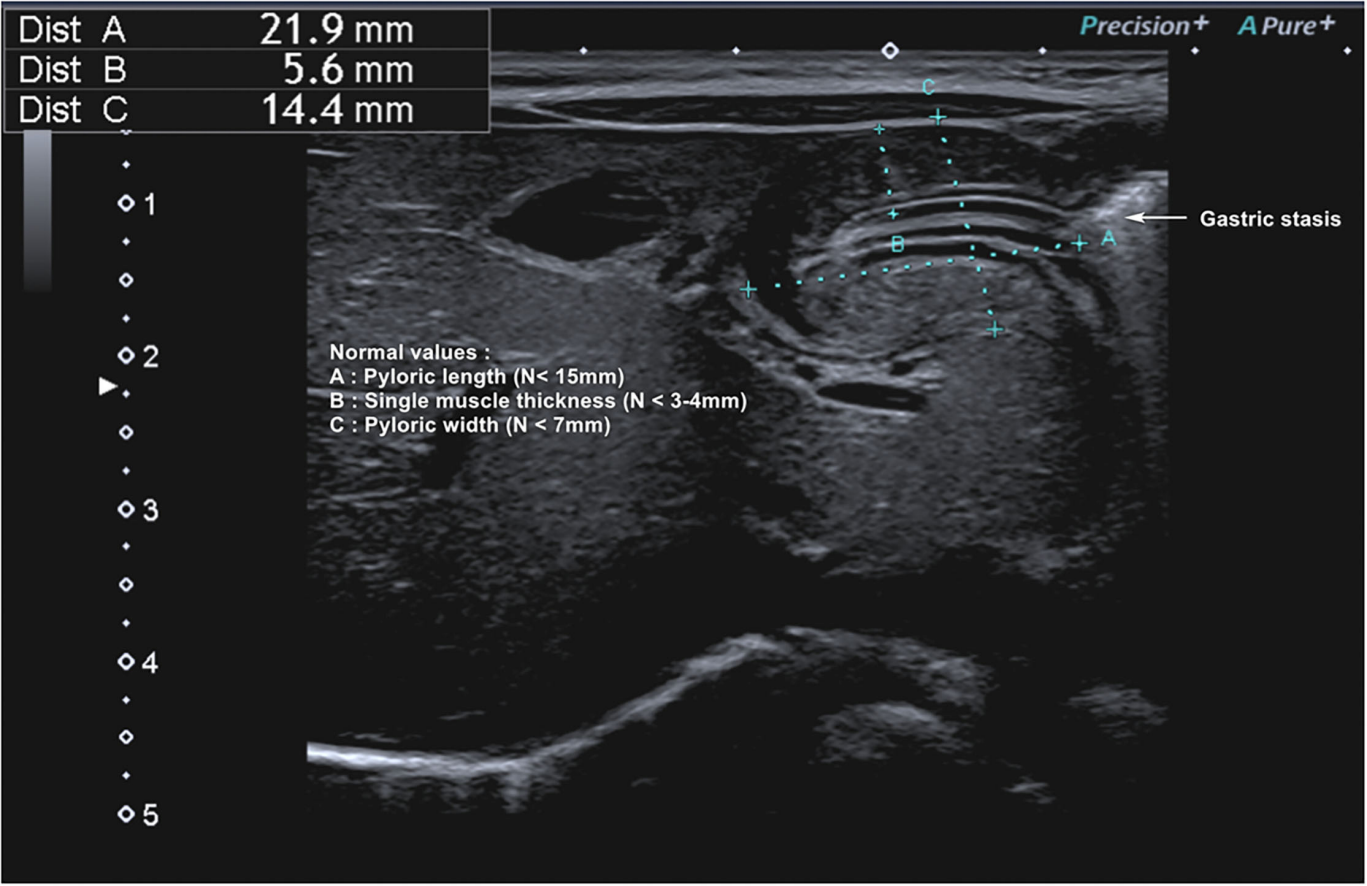
Hypertrophic pyloric stenosis. Gastric POCUS consisted of the measurements of pylorus muscle thickness and length, and HPS diagnosis was confirmed if they were >3 and 15 mm, respectively. A 6–10 MHz linear probe in a transverse position allows identifying the gallbladder in the supine position. The pylorus is usually located slightly medial and posterior in relation to the gallbladder.

Totally, eight studies were identified (Table 4) (5, 12, 67, 69–72, 80), and 652 infants younger than 6 months presenting with signs of HPS were recruited in the ED; three studies examined gastric POCUS performed by surgeons (5, 67, 69) and five by ED physicians (12, 70–72, 80). In these studies, POCUS was compared to RADUS; in four other studies, the learning curve and training were assessed (surgeons training surgeons, surgeons training ED physicians, or ED physicians training ED physicians) (69, 71, 72). Figure 6 presents an example of HPS and details the gastric POCUS technique. Regarding the accuracy of POCUS compared to RADUS, sensitivity and specificity ranged from 96.6 to 100% and from 94 to 100%, respectively (12, 70). The measurements of the pylorus muscle obtained by radiologists or surgeons/ED physicians did not significantly differ. One study assessed the impact of POCUS on the length of ED stays and found that POCUS significantly shortened ED stay in children with HPS and time to disposition in children with no HPS (12). The ability of healthcare professionals trained in POCUS to train their ED colleagues was good, with no false positives or false negatives in two studies (69, 72), and as shown the third study, pre-/post-training test scores improved from a mean of 61–83% correct, respectively (71).

**Table 4:** Study characteristics and findings: hypertrophic pyloric stenosis.

**References**	**Study design**	**Patient characteristics**	**Gastric POCUS question**	**Intervention**	**Key findings**
Bonasso et al. (5)	Review	Four studies conducted in infants	Hypertrophic pyloric stenosis	Narrative Review of the literature	Accuracy of POCUS performed by surgeons compared to radiologists; teachable to surgeon fellows and ED fellows; allows direct and accurate decision for surgery
Boneti et al. (67)	Prospective cohort	30 infants in ED	Hypertrophic pyloric stenosis	POCUS performed by surgeon compared to RADUS	No false-negative or false –positive results. No statistically significant difference between surgeon and radiology measurements about pyloric muscle thickness (*P* = 0.825, mean deviation = 0.4 mm) or channel length (*P* = 0.74, mean deviation = 2.2 mm).
Malcolm et al. (68)	Case series	8 infants in ED	Hypertrophic pyloric stenosis	Comparison of POCUS (ED physician) and RADUS	HPS was visualized by ED physicians on ultrasound either immediately upon scanning or within a few minutes shortly afterward. All these cases were confirmed by subsequent Radiology Department ultrasound and at surgery
McVay et al. (69)	Prospective cohort	71 infants in ED	Hypertrophic pyloric stenosis	POCUS training (surgeon resident training surgeon resident) and confirmation by RADUS	No false-negative or false –positive results. No statistical difference between the radiology department and fellow measurement when evaluating muscle width or channel Length
Park et al. (12)	Retrospective cohort	130 infants in ED	Hypertrophic pyloric stenosis	Comparison of POCUS (ED physicians) and RADUS and POCUS+RADUS	POCUS showed a sensitivity of 96.6% and specificity of 94.0%. Length of stay in the ED (EDLOS) was shorter in the POCUS-performed group than in the RADUS-only group (2.6 vs. 3.8 h, *P* = 0.015). Among non-HPS patients, time to disposition (1.8 vs. 2.7 h, *P* = 0.005) and EDLOS (2.0 vs. 3.0 h, *P* = 0.004) were shorter in the POCUS-performed group than in the RADUS-only group. Performing POCUS followed by RADUS did not significantly delay the treatment among HPS patients
Sivitz et al. (70)	Prospective cohort	67 infants in ED	Hypertrophic pyloric stenosis	POCUS (ED physicians) compared to RADUS	Pediatric EPs correctly identified all 10 positive cases, with a sensitivity of 100% [95% confidence interval (CI) = 62–100%] and specificity of 100% (95% CI = 92–100%). No statistical difference between the measurements obtained by pediatric EPs and radiology staff for pyloric muscle width or length (*p* = 0.5 and *p* = 0.79, respectively).
Tejwani et al. (71)	Prospective cohort	329 infants in ED	Hypertrophic pyloric stenosis	POCUS performed by ED physicians compared to RADUS (learning curve)	Fellows showed a significant improvement between the training scans and deciles 3, 4, and 5 (*p* < 0.05). Pre/post-training test scores showed improvement from a mean of 61–83% correct, respectively.
Wyrick et al. (72)	Prospective cohort	17 infants in ED	Hypertrophic pyloric stenosis	POCUS training (surgeon resident training pediatric ED resident) and confirmation by RADUS	No false-negative or false –positive results. No statistical difference between the radiology department and fellow measurement when evaluating muscle width (*p* $\frac{1}{4}$ 0.21, mean deviation $\frac{1}{4}$ 0.2 mm) or channel Length (*p* $\frac{1}{4}$ 0.47, mean deviation $\frac{1}{4}$ 0.6 mm).

*POCUS, point-of-care ultrasound; RADUS, radiologist ultrasound; CSA, cross-sectional area; ED, emergency department; HSP, hypertrophic pyloric stenosis*.

## Other Indications: Ventilatory Support

Totally, four randomized controlled trials (73–76) were conducted in a total of 337 children undergoing general anesthesia for elective surgery (Table 5). Different ventilation modes and methods were assessed, and POCUS was used as a primary or secondary outcome to assess gastric insufflation induced by ventilatory support (face mask vs. ventilator, different positive inspiratory pressure (PIP) levels, manual vs. pressure-controlled face mask). The technique was similar to the one described in Figure 3. POCUS was performed by trained anesthesiologists and aimed to measure the gastric antrum CSA as a surrogate of gastric insufflation. One study (74) compared these measurements with gastric auscultation and showed that all cases of gastric insufflation detected by auscultation were also detected by US, although not vice versa. Gastric POCUS helped determine the optimal level of positive inspiratory pressure (PIP) and suggested that pressure-controlled face masks limited gastric insufflation.

**Table 5:** Study characteristics and findings: gastric insufflation/mechanical ventilation.

**References**	**Study design**	**Patient characteristics**	**Gastric POCUS question**	**Intervention**	**Key findings**
Qian et al. (73)	RCT	84 children in operating room	Ventilation support impact	To identify the best PIP for providing adequate ventilation and minimal gastric insufflation. Incidence of gastric insufflation—assessed with POCUS (anesthesiologist) before and after 120 s of mask-ventilator ventilation with various levels of PIP.	An inspiratory pressure of 12 cm H_2_O was sufficient to provide adequate ventilation with a lower occurrence of gastric insufflation. Gastric insufflation was detected in 32 children using ultrasonography (3/18 in group P8, 5/18 in group P10, 7/18 in group P12, 8/16 in group P14, and 9/14 (64%) in group P16). There were statistically increases in the antral CSA in subgroups P14 GI+ and P16 GI+ ('statistically significant rise in gastric insufflation above 12 cm H_2_O)
Park et al. (74)	RCT	48 children in operating room	Ventilation support impact	The primary outcome was to compare the PAP (peak airway pressure) during facemask ventilation with MV and PCV. Incidence of gastric insufflation—assessed for 3 min when patient was ventilated with one of two methods, insufflation assessed using simultaneously by POCUS (anesthetist) and auscultation	PAP did not show any difference between patients with and without gastric insufflation detected by US. Gastric insufflation was detected in 10 children by US (7/23 in Group MV vs. 3/22 in Group PCV, *P* = 0.284) and in 5 by auscultation (3/23 in Group MV vs. 2/22 in Group PCV, *P* > 0.999). All cases detected by auscultation were also detected by US, although not vice versa. Overall Gastric antral area was expanded after facemask ventilation compared with the pre-ventilation values in both groups, although intergroup differences were not observed [Group MV, 73 (59.4–115.9) vs. 67 (50.3–99.1) mm^2^ (*P* = 0.329); Group PCV, 111 (69.1–199.9) mm^2^ vs. 94 (61.9–126.4) mm^2^ (*P* = 0.276)]. After facemask ventilation, the Gastric antral area of children with gastric insufflation was profoundly larger than the one of children without gastric insufflation [1.89 (1.40–2.66) mm^2^ vs. 0.82 (0.62–1.16) mm^2^, *P* < 0.001], whereas the pre-ventilation values were similar [0.71 (0.57–1.13) mm^2^ vs. 0.69 (0.52–1.10) mm^2^].”
Li and Hu (75)	RCT	34 Children in operating room	Ventilation support impact	Incidence of gastric insufflation during ventilator + mask pressure-controlled ventilation using three levels of PIP: 8, 12, 16 cm H_2_O. US performed before and after 120 s of ventilation.	After facemask ventilation for 120 s, gastric insufflation was detected in 24 children (45.3%) and the antral CSA was significantly increased in groups P12 and P16.
Lee et al. (76)	RCT	151 children in operating room	Ventilation support impact	Incidence of gastric insufflation—assessed during 90 s following paralysis, when patient was ventilated with one of two methods, insufflation assessed by POCUS (anesthetist) and compared to auscultation	The incidence of gastric insufflation was significantly higher in the MV group than in the PCV group [48 vs. 12%, respectively; odds ratio (OR) 7.78, 95% confidence interval (CI) 3.38-17.9; *P* < 0.001]. Ultrasonography detected gastric insufflation more sensitively than did epigastric auscultation; gastric insufflation was detected by ultrasonography in 21, by auscultation in 10, and by both methods in 17 patients. There was no significant difference in the baseline gastric antral area between the two groups. However, the post-ventilation gastric antral area was significantly larger in the MV group [1.3 (0.6) cm^2^) than in the PCV group [1.0 (0.5) cm^2^; 95% CI of differences, 0.13-0.46 cm^2^; *P* = 0.001].

*POCUS, point-of-care ultrasound; RADUS, radiologist ultrasound; CSA, cross-sectional area; RCT, randomized controlled trial. FB, foreign body*.

## Discussion

This scoping review found that gastric POCUS was assessed or used in a significant number of clinical scenarios in pediatrics. However, most commonly, it was used to assess gastric contents/volume in the peri-operative setting. We did not find any studies conducted in the PICU, but a few in neonatal intensive care. Most studies were recent (published in the last 10 years) as the availability of portable ultrasound machines was not common before the 2010's, when radiologists used to be responsible for ultrasounding patients. POCUS has been shown to decrease the time to diagnosis and treatment (12). Overall, the gastric POCUS learning curve is short, and several studies showed good reliability of measurements and interpretations (7–9, 12, 52, 64, 69, 70, 72). Gastric POCUS is likely to increase in different pediatric clinical settings in future. However, POCUS may not always be cost- or time-efficient, especially in elective sedation/anesthesia considering the low prevalence of a full stomach in this setting (40, 42, 49, 81).

### Review Findings and Limitations

#### Gastric Emptying/Content Evaluation Studies

Different techniques (and mathematical extrapolation of gastric volume from the surrogate antrum or cardia length measurements) have been proposed. Even if the spheroid calculation of the stomach appears the most accurate (43–46), the use of the antrum CSA has been used for decades in adults and is easier to perform (7, 52), especially when monitoring is required. Mathematical models to calculate gastric volume compared to NGT aspiration showed study design limitations and produced rather inaccurate models, preventing their use in clinical practice (35). So, gastric ultrasound may be useful to estimate the gastric content and volume status, mainly based on qualitative assessment, possibly completed by gastric fluid volume calculation (82). The use of gastric POCUS to assess gastric emptiness prior to sedation/anesthesia may limit the risk of aspiration during induction (with increased morbidity and mortality rates). But considering the low prevalence of a full stomach after recommended fasting times, this might be better to be applied to emergency sedation/anesthesia, rather than all children systematically (2, 6, 7, 82). However, it would allow for a fortuitous diagnosis of full stomach (22, 40, 42).

#### Impact of Feed Type on Gastric Emptying

Inconsistent results were found among these studies (18, 28, 33, 38, 44–46, 57), thus questioning the use of gastric emptying time as a clinically relevant surrogate for assessing feeding tolerance. However, these studies allowed for reducing fasting times recommended in recent guidelines: breast milk feeds, fortified or not, are encouraged until 3 h before anesthesia induction (82, 83).

#### Intragastric Foreign Bodies

Gastric POCUS was useful and limited the use of conventional radiography and irradiation in children. However, all studies were low-quality case series, and larger studies are required to better define the role of gastric POCUS in this setting.

#### NGT Placement

Blind insertions of the NGT can be sub-optimal in adult and pediatric patients (84). The risk of NGT malposition is increased in certain populations including critically ill patients, which may lead to complications such as gastric perforation, placement within the tracheobronchial tree, aspiration, or pneumothorax (85). Gastric POCUS can help identify the correct NGT placement and may reduce the need for ionizing radiation (x-ray) to confirm position (8, 9, 86, 87), which currently remains the gold standard method. Of the four studies (excluding the case report), two included only preterms and newborns. One study conducted in children lacked the gold standard abdominal x ray comparison (65). However, of the studies that report sensitivity, this was high to very high (88–98%) with good positive predictive values. No study was able to calculate specificity. The injection of an air bolus in the NGT (creates “bubbles” in the gastric fluid) may help confirm the correct placement of the tube as these bubbles are more easily detected during POCUS assessment. As NGT misplacement in the lower airways may have catastrophic consequences, the gold standard assessment for correct NGT placement is essential when gastric POCUS fails to confirm correct the intragastric position. In addition, as NGTs are often placed in the gastric fundus, gastric POCUS will neither consist of a sole antrum assessment nor fundus exploration, as described in Figure 5. Further robustly undertaken blinded studies are required in children beyond newborns and would be of value in the acutely and critically ill pediatric population.

#### Hypertrophic Pyloric Stenosis

Gastric POCUS appeared reliable compared to RADUS, and this technique could be implemented more broadly in the ED to reduce time diagnosing HSP and allowing patient care or discharge.

#### Optimizing Ventilatory Support

Totally, four high-quality studies (RCTs) used, rather than assessed, gastric POCUS in children receiving ventilation support, to evaluate gastric insufflation. However, the validation of this technique has not been clearly established in this setting.

Recent guidelines on the use of POCUS in pediatrics rarely mention gastric POCUS. In critically ill children (European guidelines published in 2020 on POCUS for critically ill children and neonates), cardiac, lung, cerebral, and vascular line placement and abdominal POCUS use are well-detailed (88). For abdominal POCUS, guidelines focused on intra-abdominal fluid detection and drainage or aspiration, parenchymal changes of solid organs and urinary obstruction, and peristalsis assessment and necrotizing enterocolitis detection only. Gastric POCUS was mentioned only for HPS detection. Recent neonatal guidelines (89) mention the use of abdominal POCUS to detect necrotizing enterocolitis, gut dysmotility, or anuria, but again gastric POCUS is not mentioned. The most recent European anesthesia guidelines (82) do discuss the benefits of using gastric POCUS in children when compliance with fasting instructions is unsure or in case of emergency anesthesia. Some recent pediatric emergency POCUS reviews (90, 91) have also mentioned gastric POCUS, and O'Brien et al. rated it as probably useful to identify foreign bodies and HSP, respectively. Other published reviews have not mentioned gastric POCUS (92, 93).

An expert group of adult and pediatric physicians has recently created a website dedicated to gastric POCUS, which describes the technique and presents a few examples of findings in various clinical settings (94). It provides useful information to strengthen clinicians' knowledge about gastric POCUS.

Most studies reported gastric POCUS performed by physicians (ED, intensivists, pediatricians, surgeons, anesthesiologists), but its use could be extended to other healthcare professionals like specialist nurses and advanced nurse practitioners who spend more time at the bedside (especially in the PICU and NICU), and manage NGT placement, and also potentially assess stomach volume and reduce fasting times for common planned procedures. Indeed, nurse-led urinary output algorithms have already been published using POCUS bladder scanning (95).

We did not find any study published in the PICU setting. However, two studies using gastric POCUS in the PICU are currently registered on www.clinicaltrial.gov: the GastriPed study (NCT04119089) aims to compare gastric residual measurements performed by NGT aspiration and gastric POCUS; the GastrExtub study (NCT05181904) aims to monitor gastric content/volume with gastric POCUS in the peri-operative setting. Furthermore, findings presented in this scoping review could be used to implement and evaluate its use in the PICU and to utilize it more for foreign body ingestion and HSP diagnosis. Ensuring correct placement of the NGT is also crucial in critically ill children for whom recent guidelines strongly recommend early enteral nutrition (96, 97). Gastric POCUS could also help confirm gastric emptiness, which is often expected prior to procedures like extubation and procedures requiring recurrent sedation/anesthesia (burn patients). This could reduce the often prolonged fasting times and related nutrition debt, and incidence of gastric aspiration. Finally, gastric insufflation may also be problematic in children in the PICU, especially if uncuffed endotracheal tubes are used, in situations requiring ventilatory pressures or high-frequency oscillatory ventilation or in children on non-invasive ventilation in whom gastric air distension sometimes may compromise ventilation and/or enteral nutrition efficiency. Thus, gastric POCUS may also help in optimizing ventilatory support. Ideally, the implementation of gastric POCUS in the PICU should undergo further robust evaluation studies (98).

## Conclusion

POCUS use is currently increasing in a variety of pediatric settings, including the ED, NICU, and PICU. Gastric POCUS has been validated in other clinical situations and is used prior to sedation or anesthesia and in the ED. The implementation of gastric POCUS in the PICU setting appears beneficial but requires further robust studies and closer scrutiny.

## Author Contributions

FV, LT, and CJ designed the study. FV, LT, CJ, PA, WA, CM, TN, AR, EC, and LB reviewed the study abstracts and full texts. FV, LT, PA, CM, TN, EC, and LB extracted and analyzed the data. FV, LT, CM, TN, and LB wrote a synthesis of the findings per sub-questions. EC and AR provided illustrations of gastric POCUS. FV wrote the draft manuscript which was reviewed and approved by all authors. LT English-edited the manuscript. All authors contributed to the article and approved the submitted version.

## Conflict of Interest

The authors declare that the research was conducted in the absence of any commercial or financial relationships that could be construed as a potential conflict of interest.

## Publisher's Note

All claims expressed in this article are solely those of the authors and do not necessarily represent those of their affiliated organizations, or those of the publisher, the editors and the reviewers. Any product that may be evaluated in this article, or claim that may be made by its manufacturer, is not guaranteed or endorsed by the publisher.

## Supplementary Material

Supplementary Material 1 Search equations.

Supplementary Material 2 Long rational of gastric emptying/content.
