# Supplementary material for: Gastric Point-of-Care Ultrasound in Acutely and Critically Ill Children (POCUS-ped): A Scoping Review
Source: Front Pediatr. 2022 Jul 6;10:921863. doi: 10.3389/fped.2022.921863 (PMC9298849; doi:10.3389/fped.2022.921863)
Supplement: Supplementary Material 1 — Search equations. [file Data_Sheet_1.docx]

Scoping review: gastric POCUS in pediatrics

Search equations used in three databases

**Pubmed :**

("Stomach/diagnostic imaging"[Mesh] OR (ultrasonography* [Mesh]) AND stomach* [Mesh]) AND (Infant* [Mesh] OR Child* [Mesh] OR Adolescent* [Mesh]) Filters: English, French Sort by: Most Recent

**Web of Science :**

(ALL=(neonat* OR newborn* OR prematur* OR infant* OR child* OR adolescent*)) AND ALL=(gastric ultrasonography OR gastric ultrasound OR (stomach* AND imag*) OR (stomach* AND ultrasound*))

**Embase :**

('infant'/exp OR adolescent'/exp OR 'child'/exp) AND ('ultrasound'/exp OR 'echography'/exp) AND 'stomach'/exp

Pubmed: 1437 results

Web of Science: 1239 results

Embase: 990 results

Total: 3666 results

After duplicate removal: 3231
