# Supplementary material for: Gastric Point-of-Care Ultrasound in Acutely and Critically Ill Children (POCUS-ped): A Scoping Review
Source: Front Pediatr. 2022 Jul 6;10:921863. doi: 10.3389/fped.2022.921863 (PMC9298849; doi:10.3389/fped.2022.921863)
Supplement: Supplementary Material 2 — Long rational of gastric emptying/content. [file Data_Sheet_2.docx]

**Gastric ultrasound to assess gastric volume content and gastric emptying**

47 studies were identified

These studies aimed

- to assess gastric emptying of different amounts or types of breast milk (fortifier or not) or breakfast in infants (n = 9 cohort studies, total: 284 infants + 1 RCT with 60 infants) or children (n = 2 cohort studies, 54 children + 5 RCT, 183 children)

- to assess whether gastric ultrasound could be useful to determine gastric content volume in infants (n = 2 studies, 216 infants) or children (n = 8 studies, 463 children). Two studies described mathematical model for predicting gastric content volume in the child (Spencer et al. 2015, n = 100 children, gold standard=gastric endoscopy, and Schmitz et al. 2016, n=16 children, gold standard=MRI) and in infants (1 study, Kim et al. 2021, 192 infants, gold standard: gastric suctioning through nasogastric tube) (1–3). Another study proposed a mathematical model that applied in infants with HPS (Gagey et al. 2018: 34 infants, comparator: gastric suctioning through NG tube)(4).

- to determine the gastric content volume according to fasting duration, or in the setting of elective or emergency surgery in children (n = 11 studies, 981 children), or to assess whether the stomach had been emptied after gastric tube aspiration in infants with hypertrophic stenosis (1 study, 34 infants).

- to assess whether ENT surgery was associated with change in gastric content volume (1 cohort study in 66 children) (5)

**Techniques:**

Curvilinear low frequency (2-5 MHz) or high frequency linear transducers were used for examination. A secondary analysis of a study that assessed the relationship between the antral cross-sectional area and the aspirated volume of gastric contents under endoscopy focused on whether the use of linear transducer provided better view of the gastric antrum than the curvilinear curve in children (6) and found that curvilinear transducer was associated with the best view in the older children (aged 47 vs 26 months and weighing 47 vs 25 kg). The gastric antrum was scanned in the epigastric sagittal plane, either in the supine or in the right lateral decubitus position or in both positions, for qualitative assessment and/or for the measurement of the antral cross-sectional area. In some studies, longitudinal scan of the stomach was performed, allowing measurement of 3 diameters (anteroposterior, transverse and longitudinal axes) for calculation of the spheroid stomach volume (7–9). Gastric emptying was assessed using repeated measurements of the antral cross-sectional area or of spheroid stomach volume calculation. Gastric content volume was calculated in 4 studies using the mathematical model by Spencer et al. (R² = 0.60) and in one study, it was calculated using the mathematical model by Schmitz et al. (R² = 0.582).

**Main results**

****Gastric ultrasound as a tool for estimating gastric content volume***

-In a study performed in 24 preterm infants, Perrella et al (2013) have found that the spheroid calculation of the stomach volume was the most reliable measure of stomach volume; these authors used this technique in their studies (8–10). The measurement of the antral cross-sectional area is easy to perform and has been described in adults for decades to assess gastric emptying through assessing the change in area over time. In children aged > 1 year, several studies reported a significant correlation between antral area and gastric volume (r² = 0.56 in the supine position) that was improved when ultrasound examination was performed in the right lateral decubitus position (1–3,11–15). Cut-off values of antral area of 219 mm² in the supine position and 307 mm² in the right lateral decubitus position allowed discriminate between empty or full stomach, with a sensitivity of 75% and a specificity of 36% in the supine position, and a sensitivity of 76% and a specificity of 67% in the right lateral decubitus position, in children aged > 1 yr (13,14). Spencer et al. (2015) and Schmitz et al. (2016) described mathematical models for predicting gastric content volume in the child (Spencer 2015, comparator=gastric endoscopy, and Schmitz 2016, comparator=MRI) and Kim et al (2021) described a mathematical model that could be applier in infants (comparator: gastric suctioning through nasogastric tube) (1–3). Another study proposed a mathematical model that applied in infants with HPS (34 infants, comparator: gastric suctioning through NGtube) (4). These studies did have some limitations and produced rather inaccurate mathematical models, preventing their use in clinical practice. Qualitative assessment based on the visualization of fluid content in the supine and in the right lateral decubitus position has also been described in the adult, with a 0-2 qualitative grading scale that correlated well with predicted gastric volume (16). In children aged 12 months-17 years, Spencer et al. (1) reported that Grade 0 antrum (empty antrum in both position) was associated with gastric fluid volume of 0.3 ml/kg, significantly lower than this associated with Grade 1 antrum (fluid content seen in the right lateral decubitus position) and the Grade 2 antrum (fluid content seen in both the supine and the right lateral decubitus position) that was associated with the highest gastric fluid volume (1.5 ml/kg). So, gastric ultrasound may be a useful tool for estimating gastric content status, mainly based on qualitative assessment, possibly completed by gastric fluid volume calculation, as stated in the recent European guidelines for preoperative fasting in children (17).

****Gastric emptying***

The repeated measurement of antral area or of spheroid stomach volume calculation may be used to assess gastric emptying in the preterm infant, the neonate and older children. Applications include measuring feeding tolerance in neonates and assessing preoperative fasting or aspiration risk.

Several studies investigated whether the composition of various formula feeds in neonates had any impact on gastric emptying (thickened formula and energy or protein enriched formula and expressed breast milk). Two studies (10,18) found that fortified and thickened formula did affect gastric emptying, though the clinical relevance of these findings remained questionable. Gathwala et al. (19) did not find delayed gastric emptying in when fortifying expressed breast milk in preterm infants, and Fabiani et al. (20) did not find any significant difference regarding the gastric emptying time between standard and fortified formula in 47 infants. Yigit et al. (21) also reported no significant difference in gastric emptying rate in low-birth-weight infants receiving breast milk, half-fortified formula and fully fortified formula. Gastric emptying may also be influenced by infant positioning. In contrast, volume of enteral feeding did not affect gastric emptying (8,9). Interestingly, Baldassare et al. (22) did not find any significant relationship between gastric emptying time and days to achievement of full enteral feeding in preterm infants randomly assigned to receive for the first 14 feeding days either intact protein premature formula or extensively hydrolyzed protein formula, hence questioning the use of gastric emptying time as a clinically relevant surrogate for assessing feeding tolerance.

In preterm infants in NICU, Beck et al. found that the mean gastric emptying time of milk feed was less than 4 hrs (23). Lee et al. (24) assessed the gastric emptying time after formula feeding in the neonate and reported a mean time of 93 min, ranging for 45 to 150 min, without any significant difference according to the sex nor the mode of delivery. Based on these data, current fasting guidelines recommend breast milk feeding, fortified or not, be encouraged until 3 hours before anesthesia induction (17).

In children, gastric emptying time less than 4 hours has been reported after low-fat milk, breast milk and light breakfast (25–28) while it was less than 1 hour after clear fluid (29) and less than 90 min after carbohydrate rich drink (30,31). Song et al. and Bansal et al. (32,33) reported reduced gastric fluid volume 2 hrs after carbohydrate drink than after prolonged fasting, while a pilot study reported that gastric emptying of apple juice, 2% milk or high protein drink was < 4 hrs in 8-14 aged children (34). Furthermore, fluid volume (3 vs 5 ml/kg) did not affect gastric emptying in 44 children aged > 6 years (35), and gastric emptying of clear fluids was enhanced when in children positioned in a semi-seated position compared to the full supine position (36). These results all contribute to reduced fasting duration in children (17,37).

****Preoperative gastric volume***

Several studies and case series assessed the rate of full stomach or higher gastric contents in elective and emergency children. In fasting children scheduled for elective surgery, gastric content volume was low (38) and the prevalence of “stomach at risk of aspiration” was 1% in 200 children (39), while Gagey et al. (40) and Evain et al (41) reported prevalence of full stomach of 51% and 37%, respectively, in emergency patients. In children admitted in the emergency department for procedural sedation and who fasted for > 6hrs, ultrasound assessment of gastric contents was feasible in 83% to 97% of 343 children and 18% to 69% of these had a full stomach (42–44). Gagey et al. (40) reported that ultrasound guided anesthetic strategy led to 85% appropriate induction sequence technique compared to 49% after clinical assessment alone in non-elective children, while ultrasound monitored gastric suctioning allowed to perform non rapid induction sequence in 88% of 34 infants scheduled for pyloromyotomy (4). Thus, pre-induction gastric ultrasound assessment could be useful for a tailored approach when inducing children in the setting of emergency surgery. Additionally, gastric ultrasound can be helpful when a child's fasting history is unclear or they've recently consumed bowel prep solutions (17,45–47). Conversely, performing gastric ultrasound to each elective child is probably not cost-effective when considering the low prevalence of full stomach in this setting (48), though it could allow fortuitous diagnosis of full stomach (49).

****Monitoring gastric content during surgery***

In another prospective cohort study, the change in gastric content volume during ear nose and throat surgery could be assessed using gastric ultrasound. The hypothesis was that some blood could be found in the stomach during these surgeries, hence increasing the risk of postoperative aspiration. In this study, no significant change in antral area, gastric fluid volume and qualitative assessment was found, providing reassuring results for the clinician (5).

References:

1. Spencer AO, Walker AM, Yeung AK, Lardner DR, Yee K, Mulvey JM, et al. Ultrasound assessment of gastric volume in the fasted pediatric patient undergoing upper gastrointestinal endoscopy: development of a predictive model using endoscopically suctioned volumes. Paediatr Anaesth. 2015;25(3):301–8.

2. Schmitz A, Schmidt AR, Buehler PK, Schraner T, Frühauf M, Weiss M, et al. Gastric ultrasound as a preoperative bedside test for residual gastric contents volume in children. Paediatr Anaesth. 2016;26(12):1157–64.

3. Kim E-H, Yoon H-C, Lee J-H, Kim H-S, Jang Y-E, Ji S-H, et al. Prediction of gastric fluid volume by ultrasonography in infants undergoing general anaesthesia. Br J Anaesth. 2021;127(2):275–80.

4. Gagey A-C, de Queiroz Siqueira M, Desgranges F-P, Combet S, Naulin C, Chassard D, et al. Ultrasound assessment of the gastric contents for the guidance of the anaesthetic strategy in infants with hypertrophic pyloric stenosis: a prospective cohort study. Br J Anaesth. 2016;116(5):649–54.

5. Desgranges F-P, Gagey Riegel A-C, Aubergy C, de Queiroz Siqueira M, Chassard D, Bouvet L. Ultrasound assessment of gastric contents in children undergoing elective ear, nose and throat surgery: a prospective cohort study. Anaesthesia. 2017;72(11):1351–6.

6. Spencer AO, Walker AM. Antral sonography in the paediatric patient: can transducer choice affect the view? Br J Anaesth. 2015;114(6):1002–3.

7. Perrella SL, Hepworth AR, Simmer KN, Geddes DT. Validation of ultrasound methods to monitor gastric volume changes in preterm infants. J Pediatr Gastroenterol Nutr. 2013;57(6):741–9.

8. Perrella SL, Hepworth AR, Simmer KN, Hartmann PE, Geddes DT. Repeatability of gastric volume measurements and intragastric content using ultrasound in preterm infants. J Pediatr Gastroenterol Nutr. 2014;59(2):254–63.

9. Perrella SL, Hepworth AR, Gridneva Z, Simmer KN, Hartmann PE, Geddes DT. Gastric emptying of different meal volumes of identical composition in preterm infants: a time series analysis. Pediatr Res. 2018;83(4):778–83.

10. Perrella SL, Hepworth AR, Simmer KN, Geddes DT. Influences of breast milk composition on gastric emptying in preterm infants. J Pediatr Gastroenterol Nutr. 2015;60(2):264–71.

11. Fukunaga C, Sugita M, Yamamoto T. Validity of ultrasonographic measurement of gastric volume in fasted pediatric patients without sedation. J Anesth. 2016;30(5):900–3.

12. Schmitz A, Thomas S, Melanie F, Rabia L, Klaghofer R, Weiss M, et al. Ultrasonographic gastric antral area and gastric contents volume in children. Paediatr Anaesth. 2012;22(2):144–9.

13. Moser JJ, Walker AM, Spencer AO. Point-of-care paediatric gastric sonography: can antral cut-off values be used to diagnose an empty stomach? Br J Anaesth. 2017;119(5):943–7.

14. Moser JJ, Walker A, Spencer A. Can pediatric gastric sonography be used as a clinical tool to establish empty antral cross-sectional areas? Can J Anesth. 2017;64(1):S11–2.

15. Schmitz A, Kellenberger C, Weiss M, Schraner T. Ultrasonographic gastric antral area to assess gastric contents in children: comparison with total gastric fluid volume determined by magnetic resonance imaging. SWISS Med Wkly. 2010;140:15S-15S.

16. Perlas A, Davis L, Khan M, Mitsakakis N, Chan VWS. Gastric Sonography in the Fasted Surgical Patient: A Prospective Descriptive Study. Anesth Analg. 2011;113(1):93–7.

17. Frykholm P, Disma N, Andersson H, Beck C, Bouvet L, Cercueil E, et al. Pre-operative fasting in children: A guideline from the European Society of Anaesthesiology and Intensive Care. Eur J Anaesthesiol. 2022;39(1):4–25.

18. Miyazawa R, Tomomasa T, Kaneko H, Morikawa A. Effect of formula thickened with locust bean gum on gastric emptying in infants. J Paediatr Child Health. 2006;42(12):808–12.

19. Gathwala G, Shaw C, Shaw P, Yadav S, Sen J. Human milk fortification and gastric emptying in the preterm neonate. Int J Clin Pract. 2008;62(7):1039–43.

20. Fabiani E, Bolli V, Pieroni G, Corrado G, Carlucci A, De Giacomo C, et al. Effect of a water-soluble fiber (galactomannans)-enriched formula on gastric emptying time of regurgitating infants evaluated using an ultrasound technique. J Pediatr Gastroenterol Nutr. 2000;31(3):248–50.

21. Yigit S, Akgoz A, Memisoglu A, Akata D, Ziegler E. Breast milk fortification: Effect on gastric emptying. J Matern Fetal Neonatal Med. 2008;21(11):843–6.

22. Baldassarre M, Di Mauro A, Montagna O, Fanelli M, Capozza M, Wampler J, et al. Faster Gastric Emptying Is Unrelated to Feeding Success in Preterm Infants: Randomized Controlled Trial. NUTRIENTS. 2019;11(7).

23. Beck C, Marcos M, Quintero M, Roosen-Marcos M, Cladis F, Poe M, et al. Real fasting times and incidence of pulmonary aspiration in children: Results of a German prospective multicenter observational study. Pediatr Anesth. 2019;29(10):1040–5.

24. Lee JJ, Price JC, Duren A, Shertzer A, Hannum R, Akita FA, et al. Ultrasound Evaluation of Gastric Emptying Time in Healthy Term Neonates after Formula Feeding. Anesthesiology. 2021;845–51.

25. Andersson H, Frykholm P. Gastric content assessed with gastric ultrasound in paediatric patients prescribed a light breakfast prior to general anaesthesia: A prospective observational study. Paediatr Anaesth. 2019;29(12):1173–8.

26. Beck CE, Witt L, Albrecht L, Dennhardt N, Böthig D, Sümpelmann R. Ultrasound assessment of gastric emptying time after a standardised light breakfast in healthy children: A prospective observational study. Eur J Anaesthesiol. 2018;35(12):937–41.

27. Sethi AK, Chatterji C, Bhargava SK, Narang P, Tyagi A. Safe pre-operative fasting times after milk or clear fluid in children. A preliminary study using real-time ultrasound. Anaesthesia. 1999;54(1):51–9.

28. Sümpelmann AE, Sümpelmann R, Lorenz M, Eberwien I, Dennhardt N, Boethig D, et al. Ultrasound assessment of gastric emptying after breakfast in healthy preschool children. Paediatr Anaesth. 2017;27(8):816–20.

29. Beck C, Chandrakumar T, Sumpelmann R, Nickel K, Keil O, Heiderich S, et al. Ultrasound assessment of gastric emptying time after intake of clear fluids in children scheduled for general anesthesia-A prospective observational study. Pediatr Anesth. 2020;30(12):1384–9.

30. Zhang G, Huang X, Shui Y, Luo C, Zhang L. Ultrasound to guide the individual medical decision by evaluating the gastric contents and risk of aspiration: A literature review. ASIAN J Surg. 2020;43(12):1142–8.

31. Zhang Y-L, Li H, Zeng H, Li Q, Qiu L-P, Dai R-P. Ultrasonographic evaluation of gastric emptying after ingesting carbohydrate-rich drink in young children: A randomized crossover study. Paediatr Anaesth. 2020;30(5):599–606.

32. Bansal P, Saini S. A study to compare gastric volume after six hours of fasting and two hours after carbohydrate drink by ultrasonography in children undergoing elective surgery. Anesth Analg. 2021;133(3):1189–90.

33. Song I-K, Kim H-J, Lee J-H, Kim E-H, Kim J-T, Kim H-S. Ultrasound assessment of gastric volume in children after drinking carbohydrate-containing fluids. Br J Anaesth. 2016;116(4):513–7.

34. Du T, Hill L, Ding L, Towbin AJ, DeJonckheere M, Bennett P, et al. Gastric emptying for liquids of different compositions in children. Br J Anaesth. 2017;119(5):948–55.

35. Taye S, Mohammed S, Bhatia P, Kumar M, Chhabra S, Kumar R, et al. Gastric emptying time of two different quantities of clear fluids in children: A double-blinded randomized controlled study. Paediatr Anaesth. 2021;31(11):1187–93.

36. Elmetwally S, Hasanin A, Sobh L, Gohary M, Sarhan K, Ghazy D. Semi sitting position enhances gastric emptying of clear fluids in children: A randomized controlled trial. Egypt J Anaesth. 2020;36(1):170–5.

37. Thomas M, Morrison C, Newton R, Schindler E. Consensus statement on clear fluids fasting for elective pediatric general anesthesia. Pediatr Anesth. 2018;28(5):411–4.

38. Degeeter T, Demey B, Van Caelenberg E, De Baerdemaeker L, Coppens M. Prospective audit on fasting status of elective ambulatory surgery patients, correlated to gastric ultrasound. ACTA Chir Belg. 2021;

39. Bouvet L, Bellier N, Gagey-Riegel A-C, Desgranges F-P, Chassard D, De Queiroz Siqueira M. Ultrasound assessment of the prevalence of increased gastric contents and volume in elective pediatric patients: A prospective cohort study. Paediatr Anaesth. 2018;28(10):906–13.

40. Gagey A-C, de Queiroz Siqueira M, Monard C, Combet S, Cogniat B, Desgranges F-P, et al. The effect of pre-operative gastric ultrasound examination on the choice of general anaesthetic induction technique for non-elective paediatric surgery. A prospective cohort study. Anaesthesia. 2018;73(3):304–12.

41. Evain J-N, Durand Z, Dilworth K, Sintzel S, Courvoisier A, Mortamet G, et al. Assessing gastric contents in children before general anesthesia for acute extremity fracture: An ultrasound observational cohort study. J Clin Anesth [Internet]. 2022;77. Available from: https://www.embase.com/search/results?subaction=viewrecord&id=L2015640444&from=export

42. Miller A, Levy J, Krauss B, Gravel C, Vieira R, Neuman M, et al. Does Point-of-Care Gastric Ultrasound Correlate With Reported Fasting Time? Pediatr Emerg CARE. 2021;37(12):E1265–9.

43. Leviter J, Steele DW, Constantine E, Linakis JG, Amanullah S. “Full Stomach” Despite the Wait: Point-of-care Gastric Ultrasound at the Time of Procedural Sedation in the Pediatric Emergency Department. Acad Emerg Med Off J Soc Acad Emerg Med. 2019;26(7):752–60.

44. Na H, Do HH, Lee SC, Lee JH, Seo JS, Kim YW, et al. Gastric point-of-care ultrasound evaluation in pediatric emergency department procedural sedation patients; is the stomach empty at the point of scheduled revisit? Signa Vitae. 2021;17(6):59–65.

45. Boretsky K. Perioperative Point-of-Care Ultrasound in Children. Child-BASEL. 2020;7(11).

46. Parekh UR, Rajan N, Iglehart RC, McQuillan PM. Bedside ultrasound assessment of gastric content in children noncompliant with preoperative fasting guidelines: Is it time to include this in our practice? Saudi J Anaesth. 2018;12(2):318–20.

47. Azad AM, Al Madi HA, Abdull Wahab SF, Shokoohi H, Kang YJ, Liteplo AS. Gastric ultrasonography in evaluating NPO status of pediatric patients in the emergency department. Am J Emerg Med. 2019;37(2):355–6.

48. Schmitz A, Schmidt A. Can we use ultrasound examination of gastric content as a diagnostic test in clinical anaesthesia? Pediatr Anesth. 2019;29(2):112–3.

49. Munlemvo D, Moharir A, Yamaguchi Y, Khan S, Tobias J. Utility of gastric ultrasound in evaluating nil per os status in a child. SAUDI J Anaesth. 2021;15(1):46–9.
